# A new toolbox to distinguish the sources of spatial memory error

**DOI:** 10.1167/jov.20.13.6

**Published:** 2020-12-08

**Authors:** John P. Grogan, Sean J. Fallon, Nahid Zokaei, Masud Husain, Elizabeth J. Coulthard, Sanjay G. Manohar

**Affiliations:** 1 Nuffield Department of Clinical Neurosciences, University of Oxford, Oxford, UK; 2 Population Health Sciences, University of Bristol, Bristol, UK; 3 Department of Experimental Psychology, University of Oxford, Oxford, UK; 4 Oxford Centre for Human Brain Activity, University of Oxford, Oxford, UK; 5 Nuffield Department of Clinical Neurosciences, University of Oxford, Oxford, UK; 6 Department of Experimental Psychology, University of Oxford, Oxford, UK; 7 Translational Health Sciences, University of Bristol, Bristol, UK; 8 North Bristol NHS Trust, Bristol, UK; 9 Nuffield Department of Clinical Neurosciences, University of Oxford, Oxford, UK; 10 Oxford Centre for Human Brain Activity, University of Oxford, Oxford, UK

**Keywords:** visual working memory, mixture models

## Abstract

Studying the sources of errors in memory recall has proven invaluable for understanding the mechanisms of working memory (WM). While one-dimensional memory features (e.g., color, orientation) can be analyzed using existing mixture modeling toolboxes to separate the influence of imprecision, guessing, and misbinding (the tendency to confuse features that belong to different memoranda), such toolboxes are not currently available for two-dimensional spatial WM tasks.

Here we present a method to isolate sources of spatial error in tasks where participants have to report the spatial location of an item in memory, using two-dimensional mixture models. The method recovers simulated parameters well and is robust to the influence of response distributions and biases, as well as number of nontargets and trials.

To demonstrate the model, we fit data from a complex spatial WM task and show the recovered parameters correspond well with previous spatial WM findings and with recovered parameters on a one-dimensional analogue of this task, suggesting convergent validity for this two-dimensional modeling approach. Because the extra dimension allows greater separation of memoranda and responses, spatial tasks turn out to be much better for separating misbinding from imprecision and guessing than one-dimensional tasks.

Code for these models is freely available in the MemToolbox2D package and is integrated to work with the commonly used MATLAB package MemToolbox.

## Introduction

Working memory (WM) is typically measured by providing a person with a set of stimuli to remember and then probing their memory after a delay of a few seconds. For example, they may be asked which direction of one of a set of colored arrows pointed. The types of errors people make on these tasks provide important information about the processes involved in retaining information in WM over and above simply looking at accuracy or overall error (Ma, Husain, & Bays, 2014). One type of error occurs when people remember which features were presented but fail to remember the way in which they were combined, such as reporting the orientation of a different-colored arrow. This might indicate that people *misbind* the features. A second type of error involves knowing the features of an object only approximately, leading to *imprecision*. A third type of error arises if everything is forgotten and responses are essentially *guesses*. Importantly, these three types of errors may each reflect distinct processes affecting WM.

Although these processes can be distinguished when participants make binary judgments about the items in memory (Parra et al., 2010; Stark, Yassa, & Stark, 2010), recent studies have allowed greater sensitivity to these types of error by using continuous report, in which participants reproduce the feature they had to remember (Bays, Catalao, & Husain, 2009; Zokaei, Burnett Heyes, Gorgoraptis, Budhdeo, & Husain, 2015). This data-rich method allows us to separate the errors people make into their component parts. The technique of mixture modeling (Bays et al., 2009; Bays & Husain, 2008; Zhang & Luck, 2008) is now commonly used if the report domain is one-dimensional and circular, for example, line orientation or color on a wheel.

These methods have proven invaluable in understanding memory mechanisms, demonstrating, for example, that interstimulus distance affects misbinding and not precision (Emrich & Ferber, 2012), increasing set size affects misbinding and precision more than random guessing (Bays et al., 2009; Pertzov, Manohar, & Husain, 2017), and decreasing sample duration increases random guessing but not imprecision or misbinding (Bays et al., 2009). Importantly, this has also allowed mechanistic understandings in clinical populations where the different sources can be selectively impaired (Rolinski et al., 2016; Zokaei et al., 2014).

However, many working memory tasks are two-dimensional (2D; e.g., spatial location; Corsi, 1972), and extending the mixture modeling technique to work on such 2D data would allow researchers to decompose errors in their component sources. While some studies used binary report (Bays & Husain, 2008), continuous-report measures of spatial memory have emerged more recently, in which participants move a probe to its original location (Pertzov, Dong, Peich, & Husain, 2012). This can be accomplished by participants dragging or pointing to locations on a touchscreen computer, allowing precise spatial WM measures in patients with disease (Liang et al., 2016; Pertzov et al., 2013; Zokaei, Nour, et al., 2019).

To examine the types of errors made, previous analyses employed simple measures based on the distance of each response from the other items in the display (Pertzov et al., 2012). The most basic method includes all sources of errors, being simply the mean distance from the true location of the target to where a participant recalls it to have been. This distance-to-target can be compared against the distance from the response to the *nearest* stimulus that had appeared in the memory array (nearest-neighbor distance) (Pertzov et al., 2013). Such a measure removes the influence of misbinding, giving a measure of imprecision. However, this metric still conflates errors of imprecision (i.e., knowing the location only approximately) with errors due to pure guessing. Further analysis methods to take guessing into account (Pertzov et al., 2013) involve counting the number of responses that occur within a certain distance of a nontarget (swap errors), but this does not consider a person's precision or the distance between the stimuli on each trial. These simple behavioral metrics each attempt to separate out different sources of errors, but the only way to validate them has been by comparison of results with those from one-dimensional (1D) circular tasks.

One study has applied mixture modeling to 2D working memory tasks (Schneegans & Bays, 2016) and found that misbinding increased with set size, while random guessing was negligible, and that the pattern of target errors, nontarget responses, and reaction times was in keeping with a continuous-resource model, not a discrete-slot model. They also found that misbinding, but not random guessing, contributed to errors, which may highlight an advantage of spatial data; the extra dimension of separation between items leads to less overlap in response distributions centered on the items, giving greater ability to distinguish error components. This article demonstrated the usefulness of mixture modeling for spatial tasks, but to our knowledge, there is no available package for applying these models to 2D data or an investigation into the suitability and limitations of such an approach.

Here, we present 2D mixture models (adapted from a previous 1D mixture model package MemToolbox; Suchow, Brady, Fougnie, & Alvarez, 2013; MemToolbox.org) that allow us to fit a person's imprecision of location memories, along with the proportion of random guesses and nontarget responses (misbinding), taking into account stimulus locations and any biases in responses that may occur. This article serves as an introduction to the concepts behind the models, the practicalities of fitting the models, and issues that may arise when working with 2D data, and it provides examples of fitting the models to human data. The Discussion section provides an overview of the limitations and advantages of the modeling approach raised during specific tests in the Results and Method sections, and readers may consult the Contents to determine which sections are useful to them when working with these models. The new MemToolbox2D is available at https://doi.org/10.5281/zenodo.3752705 (Grogan et al., 2019).

## Method

### Model

**Figure 1.**
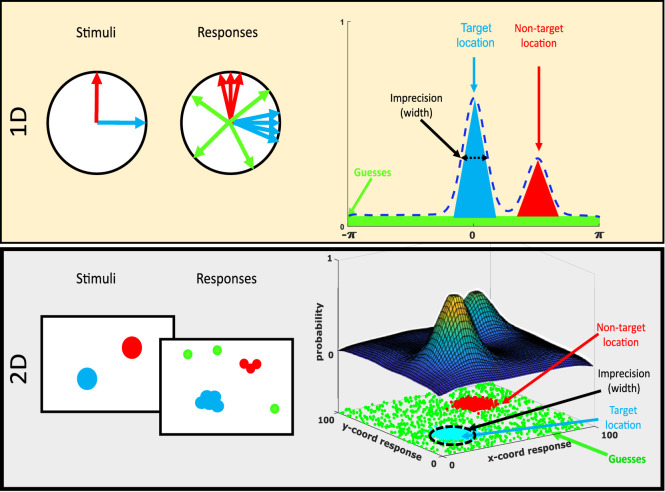
One-dimensional and 2D misbinding models. The top row shows a 1D task. Fictive stimuli and responses are shown (left) where the target (blue) and nontarget (red) were always shown at the same orientations. The responses column shows sample responses centered on the target with some imprecision (blue), around the nontarget with the same imprecision (red), and randomly distributed guesses (green). The PDF of responses (right) shows peaks around those locations with heights proportional to the number of target and nontarget responses, widths proportional to the imprecision of the memories, and a background guessing rate proportional to the height of the horizontal line. The bottom row shows a similar setup for a 2D task where the target and nontarget were always shown in the same location on a 2D screen (left), and the PDF is now 2D (right), which shows peaks of responses (upper part) around those locations (bottom part) with a 2D width (dashed circle) and uniform guessing across the entire screen (green dots).

The 1D mixture models split errors into three components (Bays et al., 2009) (Figure 1). Throughout the article, we shall focus on this three-component model (the misbinding model), although the approach is the same for related models such as the two-component mixture model (without misbinding; Zhang & Luck, 2008), and all such models have been adapted for 2D. The misbinding model has three sources of errors: imprecision, misbinding, and random guessing:

(1) $$\begin{array}{ccc} P\left(\hat{\theta }\;\right)\; & = & \;\alpha \phi_{\kappa }\left(\hat{\theta }\;-\;\theta \right)+\;\beta \frac{1}{m}\sum_{i}^{m}\phi_{\kappa }\left(\hat{\theta }\;-\;\varphi_{i}\right)\\ & & +\;\gamma \frac{1}{2\pi }, \end{array}$$

where $P(\hat{\theta }\;)$ is the probability of finding a response orientation $\hat{\theta }$, θ is the orientation of the target stimulus, φ_κ_ is the von Mises distribution (circular analogue of the Gaussian distribution), ϕ_*i*_ is the orientation of the nontarget stimulus *i*, *m* is the number of nontarget stimuli, and guessing is uniform over the entire circle (2π). The parameters α, β, γ, and κ control the proportion of target responding, nontarget responding, guessing, and the concentration of the von Mises distribution, respectively. The spread of the distributions of target and nontarget responses is assumed to be the same. As α, β, and γ must sum to 1, α is not included as a free parameter in the fitting. The three free parameters (β, γ, κ) are estimated using maximum likelihood methods.

To adapt the model for 2D data, several changes are needed. First, 2D coordinates are used in place of angles, which means that the von Mises distribution is replaced with a bivariate Gaussian distribution ψ_σ_ with standard deviation *σ* and zero covariance. Second, a distribution for the random guesses must be chosen. For simplicity, we begin by assuming that they are drawn from a uniform distribution over the entire area of the screen (*A*). This gives the following response density function:

(2) $$\begin{array}{ccc} P\;\left(\hat{\theta }\;\right) & = & \;\alpha \;\psi_{\sigma }\left(\hat{\theta }\;-\theta \right)+\;\beta \frac{1}{m}\sum_{i}^{m}\psi_{\sigma }\left(\hat{\theta }\;-\;\varphi_{i}\right)\;\\ & & +\;\gamma \frac{1}{A}, \end{array}$$

where here, **θ** and **ϕ** are vectors indicating locations on the screen, and again, α,  β, and γ sum to 1, which together with σ yield three free parameters.

The model will operate in any spatial units, as long as *A* is changed to reflect this. For the examples provided here, we will use 1366*768 as the screen area and pixels as units. The dimensions of the screen will dictate the scale of the errors in the task (i.e., on a 40 cm × 30 cm screen, the errors will be under 50 cm) and thus the scale of the imprecision parameter *σ*. The models take in a dimensions argument, which sets the screen dimensions for simulations and fitting. In other words, it implicitly sets the units for the model. Any appropriate scaling values are possible (e.g., 960*540 pixels, 46*26 cm, 30*17 visual degrees). This allows you to convert data collected on different screen sizes into standard units (e.g., visual degrees or percentage of screen size), for example, to correct for any influence that different screen sizes may have on the data. A rectangular screen is assumed, so if a different shape screen is used (e.g., a circular window on a screen), the model will need to be modified to account for this. Different dimensions can be supplied for simulating and fitting the data (e.g., if the stimuli may not appear too close to the edges of the screen but possible response locations are not so constrained). This can also be achieved by using the method described in the “Effect of stimulus separation on recovery” section below.

Previous 1D models used a circular space, meaning that responses wrapped around the domain, without any edges. In contrast, the 2D spatial tasks involve a finite space defined by the screen or screen region. When simulating the model, any responses that fall outside of the screen when drawn from the normal distributions are replaced by new samples drawn from the same distribution.

We use the framework for 1D models provided in MemToolbox (Suchow et al., 2013; MemToolbox.org) to provide a new toolbox (MemToolbox2D; Grogan et al., 2019; https://doi.org/10.5281/zenodo.3752705) for modeling 2D data that integrates with the existing 1D MemToolbox. New functions were created to run the fitting, display plots, correct for biases, and simulate the models. Thus, MemToolbox2D will not interfere with the functions of MemToolbox.

### Simulations

First, we provide simulations to show the behavior of the model during fitting and some of the issues that may impact the quality of the fits, including true parameter values, number of trials, number of nontargets, response distributions and biases, stimulus constraints, and screen edges.

For all simulations, unless otherwise specified, we simulated a task in which an agent views three stimuli. The three stimuli were randomly placed on the screen, and one was randomly chosen to be the “target,” with the other two being “nontargets.” The agent then had to place the target stimulus in the location they remembered it occurring before. This is similar to a spatial WM task used previously (Pertzov et al., 2013). We simulated 100 trials. The type of response made on each trial was determined by the parameters used for the simulations: *γ* controlled the proportion of responses that were random guesses, β the proportion that were misbinds (centered on a nontarget location), α (α = 1 – γ – β) the proportion that were target responses, and σ the imprecision (standard deviation) of target and misbinding responses. We then fit the misbinding model to these simulated data to see how accurately the true parameters could be recovered.

A large sweep of parameters was used for these simulations, ranging from almost no to almost all guessing (γ ϵ [0.01, .98]), almost no misbinding to almost all misbinding (β ϵ [0.01, 0.98]), and low to high imprecision (σ ϵ [0.1, 100]). Eleven values of each parameter were chosen, evenly spaced across these ranges, and all combinations used, with the caveat that γ and β summed to 1 or less, and we simulated each parameter combination 100 times. This parameter sweep was chosen to test how well the model can recover true parameters across a large range, including values that represent poor performance due to high guessing, misbinding, and imprecision, either in isolation or combination. These values are unlikely to occur in real data as few people will guess or misbind on all trials.

For each simulation at a particular set of parameters, the 100 simulated trials were fit by maximum likelihood estimation. The parameter fits account for the position of each of the items, as well as the response, on each trial.

To show the accuracy of parameter recovery, we plot the difference between recovered and true parameters (parameter recovery error) against the values of the true parameters; zero indicate perfect parameter recovery, and larger (positive or negative) values indicate poorer recovery. These recovery errors are plotted for each parameter, marginalized over the other parameter values.

When comparing different models or methods, we use the Bayesian information criterion (BIC; Schwarz, 1978), which adds a penalty for the number of trials and parameters to the negative log-likelihood of the model fit; smaller values indicate better-fitting models.

Code for all the simulations here is available from https://doi.org/10.5281/zenodo.3752763 (Grogan, 2019).

### Human data

To illustrate how the models work with real human data and to examine how they cope with more complex task designs, we fit previously unpublished data collected from an Ignore/Update spatial WM task (i.e., a 2D task) and compared the pattern of recovered parameters to those found on a 1D orientation analogue of this task (Fallon, Mattiesing, Dolfen, Manohar, & Husain, 2018; Fallon, Mattiesing, Muhammed, Manohar, & Husain, 2017). New data were collected from 49 healthy older adults (mean age = 71.4 years, *SD* = 5.5, 18 females, 31 males). Ethical approval was granted by South West–Central Bristol NHS REC (16/SW/0205). Participants gave written informed consent, and the study was conducted in line with the Declaration of Helsinki.

**Figure 2.**
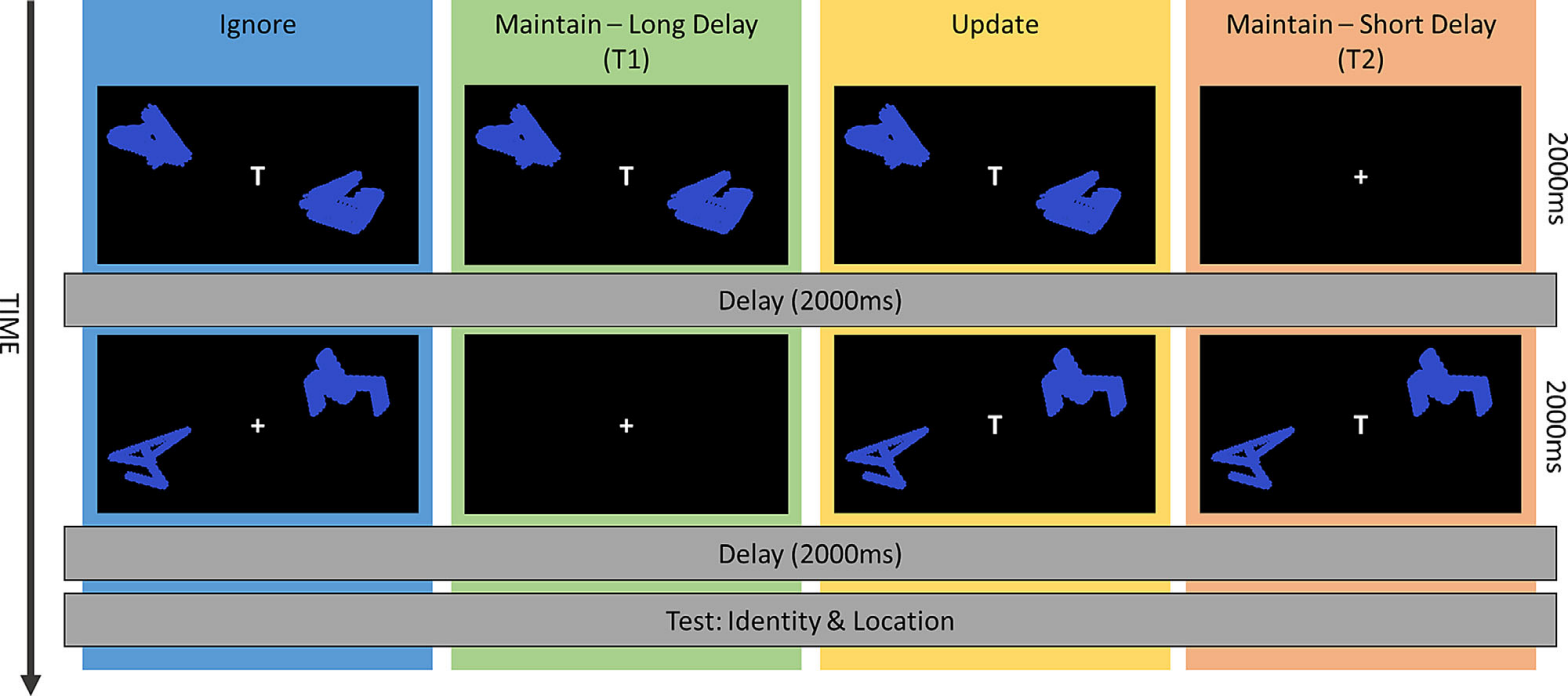
Procedure of the spatial WM task. Participants were presented a pair of test shapes (with a “T” at fixation), which could be either preceded or followed by a blank screen (Maintain T2 and T1, respectively) or by a second pair of nontest shapes (Update or Ignore, respectively). They were asked to remember the most recent pair of test shapes. At the test, one test shape and one nontest shape (novel item or distractor) were shown, and participants chose the item they remembered to have been a test shape and dragged it to where it appeared previously.

In brief, the task involved remembering a pair of abstract shapes (Figure 2) that were shown with a “T” cue in the screen center, indicating they were “test shapes”, one of which would be tested later in the trial. These could be shown at Time 1 and/or Time 2. On some trials, nontest shapes (no “T” shown in center) could be shown either at Time 2, which would not be tested, and were not to be remembered. Participants were instructed to remember the most recent test shapes they saw. They were tested with a test shape and a nontest shape (either distractor or novel shape) presented on the screen. Participants were asked first to select the shape they recalled to be in the (most recent) test shape pair by touching it, rather than the nontest shape that was presented with it. Then they were requested to drag the test shape to where they remembered it to have been shown originally.

Overall, there were four different conditions, each occurring with the same frequency (Figure 2):

■ **Ignore trials:** Consisting of test shapes at Time 1 followed by nontest shapes at Time 2
■ **Maintain long delay trials (T1):** Test shapes at Time 1 followed by a long delay
■ **Update trials:** Consisting of test shapes at Time 1 followed by new test shapes at Time 2 (so now working memory of test shapes at Time 1 had to be updated to instead store the test shapes at Time 2)
■ **Maintain short delay trials (T2):** Test shapes at Time 2 followed by a short delay.

There were two key factors in this design. First, there were irrelevant stimuli—either ones that had to be ignored (Ignore condition) or old items stored in memory that now had to be displaced by new ones (Update condition). Then there were two conditions that controlled for different maintenance durations in the Ignore and Update conditions, by simply having either a long (T1) or short (T2) delay.

Note that the ignore and update trials have three nonprobed items (one nonprobed test shape plus two distractors), while T1 and T2 trials only have one nonprobed test shape; the model accounts for this by averaging the likelihoods of misbinding to each nonprobed shape together (“*m*” in Equation 2). Other models are available that allow for two sets of nonprobed shapes, for example, with separate misbinding parameters for the distractors and the nonprobed test shape.

The task was performed on Dell laptops using a mouse, at a viewing distance of approximately 40 cm. After 11 practice trials, there were three blocks of 40 trials, giving 120 trials in total (30 per condition, random order).

## Results

Here we present several simulations showing how 2D models compare to 1D models and investigating factors influencing the accuracy of parameter recovery.

### Accuracy of recovering simulated parameters

To see what is gained from 2D responses over 1D responses, we compared how well the fitted parameters relate to the simulation parameters used to generate the simulated data. We simulated and fit 1D and 2D versions of the misbinding model. The 1D models fit the *κ* parameter for the von Mises distribution concentration, which is converted into standard deviation (*σ*) for comparison with the standard deviation parameter in the 2D models.

**Figure 3.**
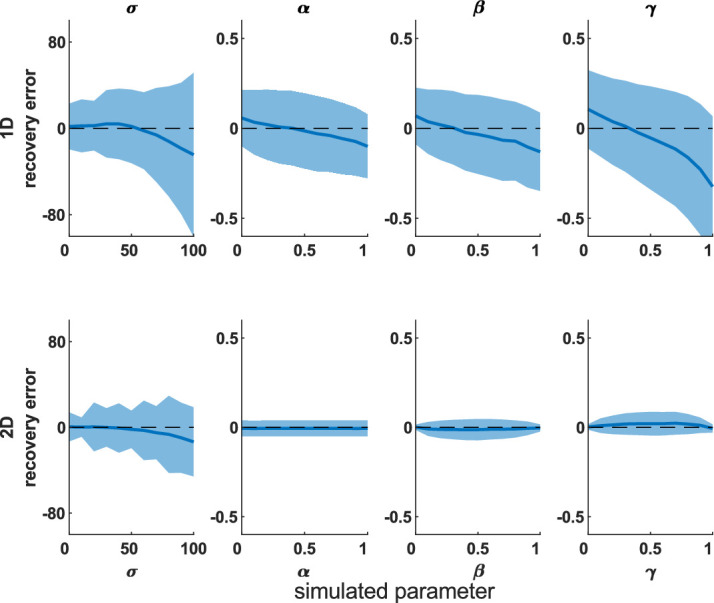
Mean difference between recovered and simulated parameters (parameter recovery error). This is shown for 1D (top row) and 2D (bottom row) misbinding models. Dashed line shows the y = 0 line, which is where there is no difference between the simulation and recovered parameters for any simulation parameter value. Shaded area shows standard deviation. Each column shows one parameter value, marginalized across all other parameters from the parameter sweep. The 2D model performs better than the 1D model (*t* tests: *p* < 0.0001; see Supplementary Figures S4–S6 for 1D models).

First, we looked for recovery error and biases, using 100 iterations across the parameter sweep. Both models recovered parameters that correlated highly with the true parameters (Table 1). Figure 3 shows the difference between simulated (true) parameters and fitted (recovered) parameters across the parameter space; the 2D model has smaller deviations from zero, meaning better parameter recovery (paired-sample *t* tests for each parameter, *p* < 0.0001).

**Table 1.** Spearman correlation coefficients between each true parameter and the recovered parameters for the 1D and 2D misbinding models. ***p < 10^−10^.

Model	σ	α	β	γ
1D	*r* = .6668***	*r* = .7831***	*r* = .7482***	*r* = .5505***
2D	*r* = .9302***	*r* = .9758***	*r* = .9706***	*r* = .9710***

We next performed a parameter sweep over β, γ, and σ, simulating each parameter set 1,000 times, and plotted the simulated and recovered parameters at various slices through this parameter space to see the effect of increasing one source of error while holding the others equal. The median recovered parameters are close to the simulated values (Supplementary Figures S1–S3 for 2D simulations and S4–S6 for 1D simulations), although there is greater deviation from the true parameters when the simulated participant generates more inaccurate responses due to higher imprecision, guessing, and misbinding.

#### Parameter trade-offs within the model

**Figure 4.**
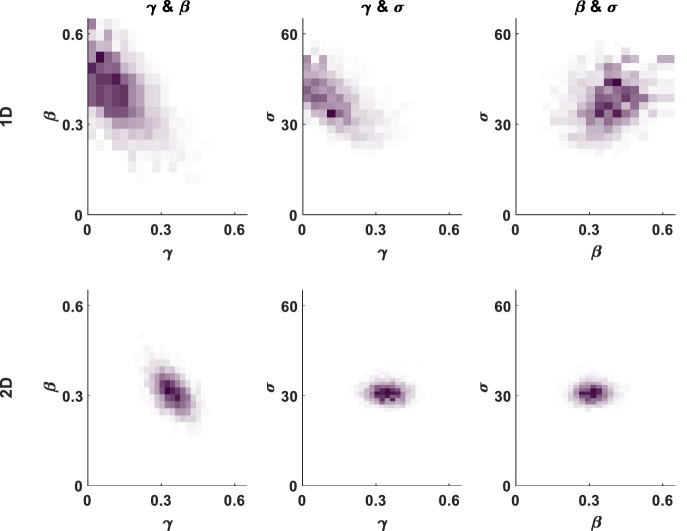
Heatmaps showing the recovered parameter trade-offs within the misbinding model in 1D (top) and 2D (bottom) space. The plots show the recovered parameter values at 10,000 MCMC posterior samples from fitting the model to simulated data (β = .3, γ = .3, σ = 30). Guessing correlates negatively with misbinding and imprecision, and misbinding and imprecision are positively correlated in both 1D and 2D, but in all cases, the correlations are weaker in the 2D model, shown here by more circular, less elliptical, patterns.

The recovered parameters show trade-offs within the 2D model, as there are within the 1D model, because responses remote from any stimuli can be explained either as random guesses or by a participant having very imprecise spatial memory. Thus, the recovered imprecision and guessing parameters negatively correlate (Figure 4) as do guessing and misbinding, but misbinding and imprecision are positively correlated. These correlations between recovered parameters are weaker in the 2D model (γ and β: *r* = –.3817, σ and γ: *r* = –.1548, β and σ: *r* = .1208; all *p* < 0.000001) than in the 1D model (γ and β: *r* = –.6799, γ and σ: *r* = –.6563, β and σ: *r* = .3314; all *p* < 0.000001). In the 1D model, imprecise responses are very likely to run into other nontargets due to the circular 1D space, whereas in the 2D model, this is less common because the locations differ in two dimensions, and the boundaries absorb imprecise responses without them wrapping around to the other side. Please note these trade-offs are within the recovered parameters, due to the dependence of them in the model, and do not mean there are trade-offs between the “true” imprecision, misbinding, and guessing that occur within people's working memory.

### Factors affecting recovery accuracy

Parameter recovery can be affected by several aspects of the task, and many of these are under the experimenter's control. Here we show how factors such as number of trials, number of nontargets, and constraints on stimulus placement affect recovery accuracy from simulations. Experimenters can thus design tasks taking these factors into account when considering their model fitting and may find it useful to simulate their tasks before running them to test their recovery accuracy.

#### Better estimates with more trials

**Figure 5.**
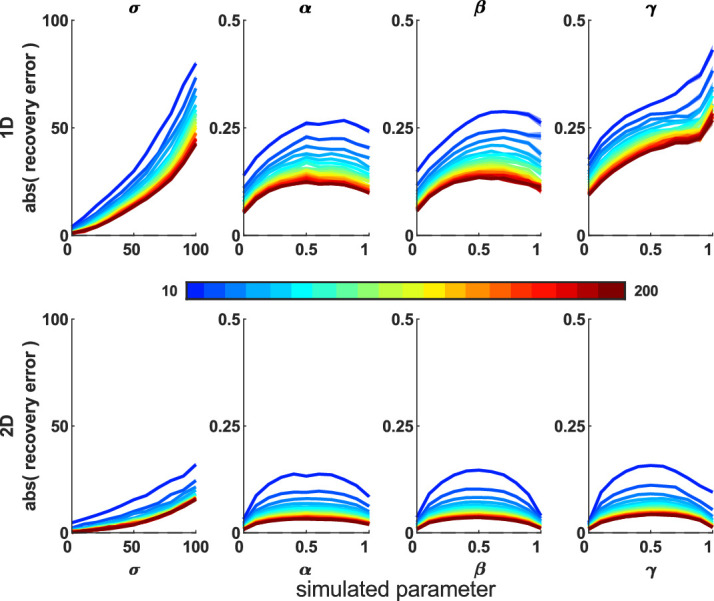
The absolute recovery error for different numbers of trials for 1D (top) and 2D (bottom) misbinding models. More trials (warmer colors—see color bar) leads to more accurate parameters (lower absolute recovery errors) in 1D and 2D models (linear regression: *p* < 10^−10^). Shading shows standard error of the mean.

The more trials available for fitting, the more accurate the parameter recovery (Figure 5). This was seen for both 1D and 2D misbinding models (linear regression: *p* < 10^−10^). As it is not always possible to have large numbers of trials, recovering simulated parameters can be useful to experimenters for assessing the accuracy of model fitting in their study.

#### More nontargets worsen parameter recovery

**Figure 6.**
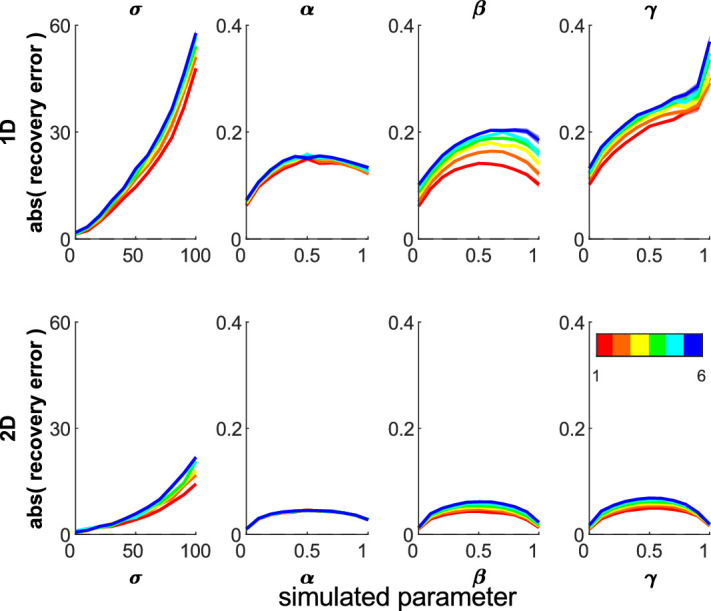
Absolute parameter recovery errors for different numbers of nontargets. More nontargets (cooler colors) leads to higher absolute recovery errors in 1D and 2D models (linear regression: *p* < 10^−10^). Shading shows standard error.

Including nontarget stimuli is useful to assess misbinding between stimuli (Bays et al., 2009). Increasing the number of nontargets (from one to six, same parameter sweep, 100 iterations) increased the error in parameter estimation (Figure 6; linear regression: *p* < 10^−10^). These differences are larger in the 1D model because with more nontargets, imprecise target responses or random guesses are more likely to end up close to a nontarget and be classed as misbinds, which acts to decrease imprecision estimates especially. In the 2D model, this is less likely as the items are separated along two dimensions and the responses do not wrap around the edges, instead being bounded by the screen edges.

#### Effect of stimulus separation on recovery

All of the 2D simulations presented here have used entirely random (uniform) generation of stimulus locations to match the 1D simulations and many 1D orientation tasks. However, many spatial WM tasks constrain the stimuli so that they cannot appear within a certain distance of other stimuli, the edges of the screen, or the center of the screen (if probe items are presented there; e.g., Pertzov et al., 2012).

**Figure 7.**
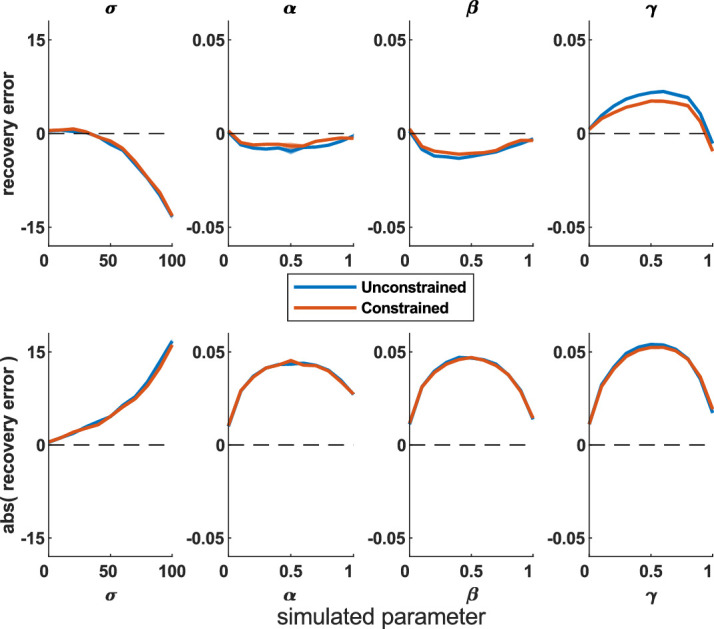
Parameter recovery errors (top) and absolute recovery errors (bottom) for the 2D model in simulated tasks with and without stimulus constraints. Adding in constraints to the stimulus locations leads to smaller errors in parameter recovery (orange line; see text for statistics). Shading shows standard error.

To examine how such constraints affect parameter recovery, we simulated two different tasks, one where stimuli appeared uniformly randomly across the entire screen (no constraints) and one where stimuli were constrained to not appear within 88 pixels of each other or the screen center, or 29 pixels from the screen edge. These distances correspond to 3 and 1 visual degrees, respectively, assuming a viewing distance of 40 cm and 42 pixels per centimeter. The entire parameter sweep was simulated 100 times for each task, which were fit with the misbinding model. The errors in the recovered parameters from the two tasks are shown in Figure 7. Paired-sample *t* tests revealed that parameters α, β, and γ had significantly smaller recovery errors when stimulus constraints were used (*p* < 10^−10^), while σ only showed a trend effect (*p* = 0.0615), but absolute recovery errors were significantly smaller for σ and γ (*p* = 0.0071, *p* = 9.6457 × 10^−8^) but not α and β (*p* = 0.4405, *p* = 0.0610). These statistics suggest that constraining stimuli can improve the recovery accuracy, although the benefits are not huge. This is likely due to the constraints reducing overlap of items (and thus responses) and making distributions centered on items less likely to be cut off by the screen edges.

### Response biases and distributions

When people perform these types of tasks, their responses may not be uniformly distributed across the screen for several reasons. First, often the stimuli themselves may not be uniformly distributed, for example, if there are areas they cannot appear in (see stimulus separation section above). Task constraints can give rise to biased distributions either through veridical memory or via strategies for guesses. Individuals may also sometimes show further biases to prefer one side of the screen, the top or the bottom half, or a radial bias where responses shrink toward the center of the screen. Many of these will depend on the experiment and stimulus characteristics, so here we shall demonstrate how two kinds of biases (constant and proportional biases) affect the model and how experimenters may account for them.

#### Constant biases

**Figure 8.**
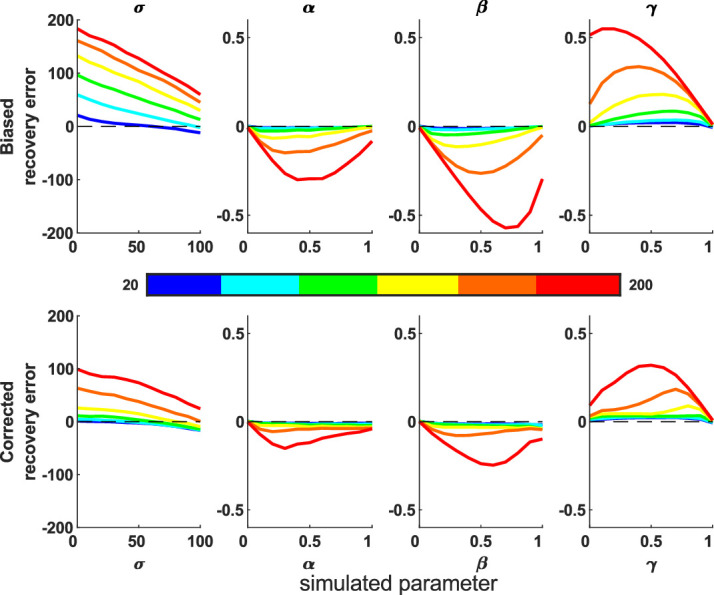
Parameter recovery errors from simulations with a constant translation bias subtracted (top) and corrected for (bottom). The color bar shows bias size (from 20 to 200 pixels). The bias correction works well for biases up to about 150 pixels in size. Shading shows standard error.

First, we simulated responses that have a constant directional bias by subtracting a fixed translation (from 20 to 200 pixels, step size = 36) to X- and Y-coordinates of all responses (and reapplying the screen boundary via edge constraining). The error of the fitted parameters rises as this bias increases (Figure 8). MemToolbox contains a function to correct for biases in 1D models, which we have adapted to work in 2D models. This fits a constant translation bias term for X- and Y-dimensions, thus improving fits as shown by smaller deviations from the true parameters (Figure 8, paired *t* tests: *p* < 10^−8^). However, when the bias is sufficiently large, responses are more likely to hit the edge of the screen (and, in this case, are edge-constrained to lie on the screen edge instead), which hinders accurate recovery.

#### Proportional biases

**Figure 9.**
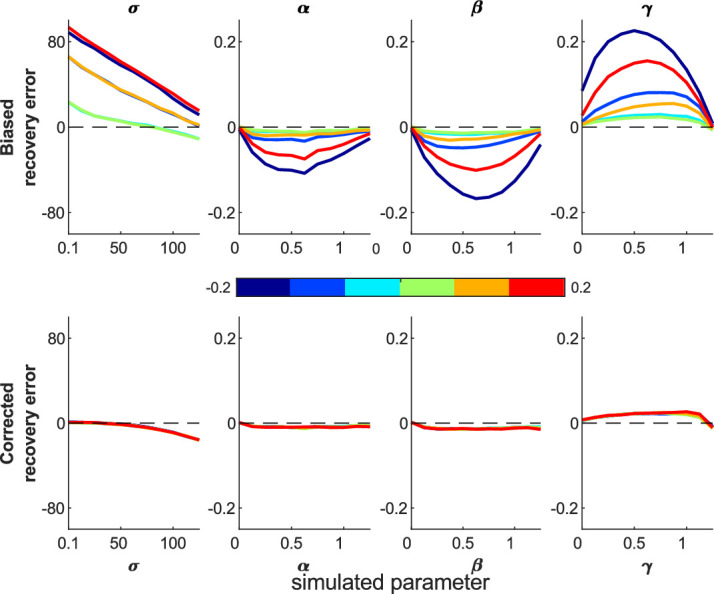
Difference plot of recovered and simulated parameter values for different proportional biases toward the right-hand screen edge. Data with (bottom) and without (top) the bias correction for different proportional biases (color bar). Positive biases mean responses move toward the right-hand screen edge, and negative biases move responses away from this edge (responses do not move vertically). The bias correction improves the fits for all parameters. Shading shows standard error.

People may be unlikely to shift all responses in the same direction by the same amount, especially if this leads to many responses landing on the screen edges, so we also simulated a proportional bias toward the right-hand edge. Here, a bias parameter controls the proportion of the distance to the edge that responses are shifted; positive values shift it toward the edge, and negative values shift away (zero means no shifting). This is perhaps more likely as a response bias in people and can also be corrected by the toolbox (Figure 9; paired *t* tests: *p* < 10^−15^).

**Figure 10.**
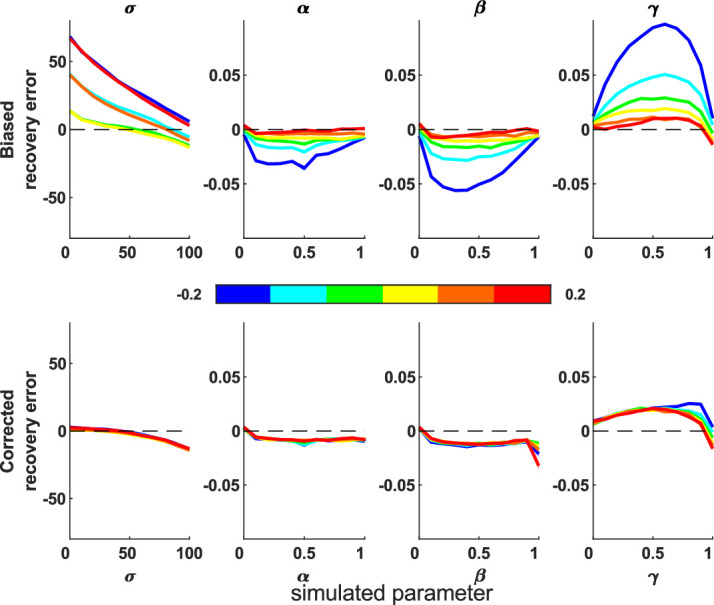
Difference plot of recovered and simulated parameter values for different radial biases. Data with (bottom) and without (top) the bias correction for different radial biases (color bar). Positive radial biases mean points move toward the center of the screen, and negative biases move points away from the center. The bias correction improves the fits for all parameters, although large guess rate parameters are not fully corrected when there is a large expanding bias. Shading shows standard error.

Next, we applied a proportional radial bias that either shrinks all responses toward the screen center (positive values) or expands them out (negative values) by a proportion of the distance the response is from the screen. Biases mainly affect the imprecision parameter (Figure 10), although the guess parameter is affected by an expanding radial bias. We adapted the bias function to account for radial biases, which improves parameter recovery (paired *t* tests: *p* < 10^−12^), especially for the imprecision parameter, although does not fully correct large guess parameters when there is a large expanding bias.

**Figure 11.**
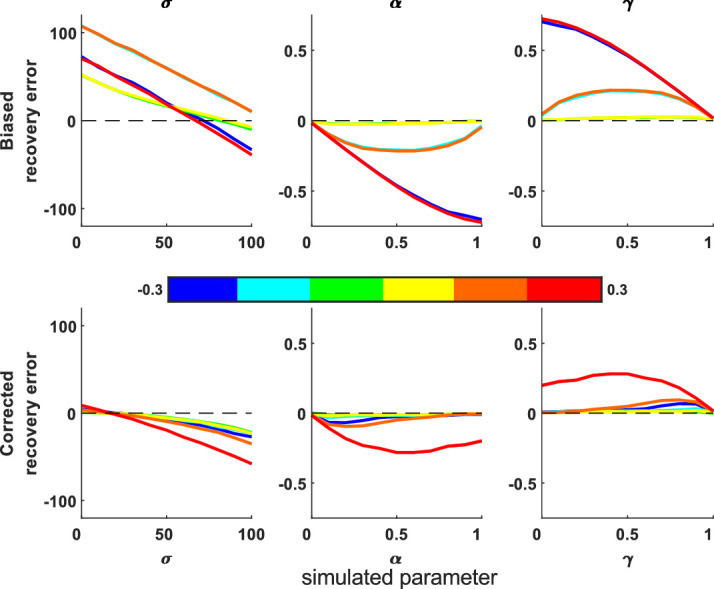
Parameter recovery errors with a radial bias toward the nontarget location on each trial. Data shown without (top) and with (bottom) bias correction. The bias correction reduced the errors in parameter recovery. This simulation had β = 0 (i.e., no misbinding). Shading shows standard error.

This function also allows us to test whether there are radial biases toward points other than the center. For instance, in trials with a single target and nontarget, it is possible that rather than the nontarget “swapping” locations with the target, it exerts a “pull” on the response locations. Note that this contribution to error is separable from misbinding, as the responses will be centered somewhere between targets and nontargets, rather than being centered on the nontarget in the case of misbinding. We can test for this by using the radial bias function and supplying the nontarget location as the bias coordinate for each trial; if we simulate such a bias, this function corrects for the bias and gives much more accurate parameter recovery (Figure 11; paired *t* tests: *p* < 10^−15^). For this simulation, we used one nontarget and the standard mixture model (i.e., no misbinding). We also fit the misbinding model to these nontarget-biased data, which did not fit the data as well as the standard mixture model with the radial nontarget bias (mean BIC = 2,720 vs. 2,437), suggesting this pattern of nontarget “pull” is distinguishable from misbinding in simulated data.

#### Response sampling for guess distributions

People may not (and indeed probably do not) have a uniform guess rate in these tasks but instead may have complex distributions that include some of the biases mentioned above but may have others we have not thought of. One way of dealing with this is to use the participants’ own responses to determine the “guessing” distribution. To achieve this, a *probability density function* (PDF) can be built from all of a participant's response locations over an experiment (across all conditions for a participant's data). Drawing from this distribution samples a response taken from a random trial, which, if the trials are independent, should be unrelated to the current trial's stimuli and thus can constitute a random guess constrained by the person's own response biases.

We implemented this in a model that uses a kernel smoothing density function to build a PDF of the person's responses over all trials, which is then used to estimate the probability of each trial's response over the entire experiment. MATLAB's ksdensity function was used to build the response PDF, which estimates the default bandwidth of the smoothing function from the data using Scott's rule. The default bandwidth can be optionally overridden to supply your own bandwidth in the response sampling function.

This model cannot easily be tested with simulations as people have nonrandom distributions of responses that we cannot predict, so we tested this model using cross-validation from the healthy participants’ data fitting (see later section or the Method section for details). We fit 90% of all the trials (combining all conditions) with the misbinding model with and without response sampling and then generated the likelihood of the responses for the remaining 10% of trials using the fitted parameters. We repeated this 100 times for each person and took the mean likelihood for each person. Crucially, the response sampling only included the 90% of training trials, not the 10% of validation trials. If the response sampling model accounts for people's response distributions and therefore gives better fits, it should lead to larger likelihoods for the out-of-sample validation trials than the uniform guessing model, whereas if it is instead simply overfitting the participants’ responses, then out-of-sample probabilities will be lower than the uniform guessing model. The response sampling model gave higher likelihoods than the uniform guessing model for the validation trials (*t* test: *p* = 4.8265 × 10^−10^), suggesting it is not simply overfitting the data but that drawing guesses from the distribution of all responses can give more accurate estimates of guess rates.

### Change detection

The models are also able to fit data from change detection tasks similar to a two-alternative forced-choice (2AFC) paradigm. Change detection tasks probe the ability to detect changes to stimuli's features, such as location. A stimulus is probed, shown displaced from its original location by some distance, and participants must decide if the location is the same or different. The paradigm is different from many 2AFC tasks used for signal detection, because often all probes have some displacement, however small, and the task is used not to measure people's ability to separate changed from unchanged items but to measure the minimum detectable changes in feature memory. These tasks give much less information on each trial about the source of error, but with sufficient data, corresponding models can still be fit. These models are adapted from the 2AFC models in the original 1D MemToolbox (Suchow et al., 2013).

The model is based on assumptions about the spatial pattern of erroneous acceptances of a displaced probe caused by the different underlying error sources. A person with perfect memory would reject all displaced probes, but as the error sources increase, they give different patterns of acceptances. Imprecision will increase acceptance closer to the target, misbinding will increase acceptance close to the nontargets, and guessing will increase acceptance uniformly. We also assume that if someone incorrectly accepts a probe of a certain displacement as unchanged, they would also have accepted smaller displacements on that particular trial. Taken together, these assumptions lead to us integrating over the continuous probability density function to estimate the probability of correctly rejecting a displaced probe, which we calculate as the probability of generating a response that is closer to the target than the current probe is. Note that this approach assumes a nonzero displacement of each item; otherwise, this integration will be affected mainly by the area on the far side of the target to the probe.

**Figure 12.**
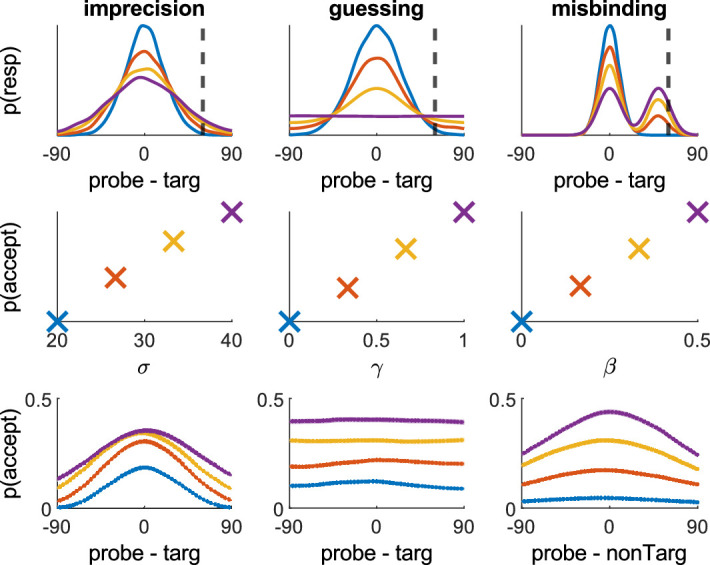
Illustrative examples of mixture modeling approach to change detection. We show 1D examples for ease, although the same principles apply for 2D (see Supplementary Figure S10). The top row shows how each error component affects the proportion of generative responses that would fall at or beyond a probed distance of 60 degrees (arbitrary y-units). The second row shows the values of the parameters used for those simulations, along with the summed probabilities of all responses that occur at ≥ 60 degrees from the target. Increasing each parameter increases the probability of incorrectly accepting this probe as unchanged. The bottom row shows the probability of accepting a probe as unchanged as a function of the probe distance (or probe-nontarget distance for misbinding). This shows that increasing imprecision increases the spread of these same responses around the target, increasing guessing affects all orientations similarly, and increasing misbinding affects responses in relation to their distance to the distractor (if we plotted probe-targ for misbinding, it would look like the guessing panel).

Figure 12 shows, when a range of different probe (and nontarget) distances is considered, different patterns of acceptance are seen: Increasing imprecision increases the acceptance of probes closer to the target, increasing guessing increases acceptance similarly across the entire circle, and increasing misbinding increases acceptance closer to nontargets.

Radial and translation biases can be included in the models as in the continuous case. As the task is change detection rather than a signal detection task (as the probe is not the same as the target, and even if it were would likely not be perceived so due to memory decay), the aim is not to measure a person's ability to separate out signals from nonsignals but rather to estimate these underlying error sources. The estimates of imprecision, guessing, and misbinding are based on the effect of probe distance on acceptance of changes. Under this framework, a person who accepts probes closer to the target does so because of less imprecise memory, and a person who accepts more probes regardless of distance does so because of increased guessing, not because of a greater propensity to accept changes. It is possible to estimate a response bias separately by averaging the proportion of acceptances.

We ran simulations and fittings for 1D and 2D versions of this. The standard mixture model (without misbinding) was used because fitting 2AFC data is slow due to the more complex probability density taking into account the nontarget locations for each trial.

**Figure 13.**
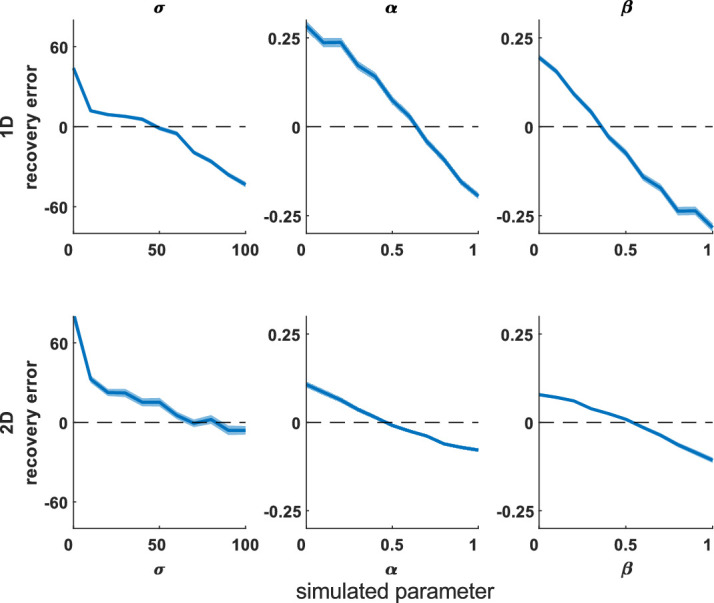
Parameter recovery errors for the 1D and 2D two-alternative forced-choice simulations. The 2D model (bottom) is more accurate than the 1D model (top), although both are far less accurate than with continuous report data (see Figure 3). Shading shows the standard error of the mean.

The parameter recovery works well in comparison to the 1D model (Figure 13), especially at higher imprecision values, but as expected is considerably less accurate than the continuous report fitting (Figure 3).

### Additional models and functions

We have developed further models, including one with a covariance parameter for the imprecision, one with separate precision parameters for targets and nontarget responses, and one with separate parameters for misbinding to two types of nontargets (e.g., the other test shape that was not probed and the nontest shapes). The latter can also be used to assess misbinding toward previous targets/responses if desired. We have also adapted all the other models present in MemToolbox (Suchow et al., 2013), including the variable precision models (Fougnie, Suchow, & Alvarez, 2012; van den Berg, Shin, Chou, George, & Ma, 2012), ensemble integration model (Brady & Alvarez, 2011), continuous resource model (Wilken & Ma, 2004), exponential decay model (Zhang & Luck, 2009), and slot model (Zhang & Luck, 2008).

We have presented only the maximum likelihood fits here, but MemToolbox2D also contains adapted functions for performing Bayesian model fitting using the Markov chain Monte Carlo (MCMC) method. These fits take longer to run, which is why we used maximum likelihood for the analyses presented here. This method also allows hierarchical model fitting across participants.

We have also adapted several other functions from MemToolbox (Suchow et al., 2013) such as the plotting functions for visualizing the data and results of the fits.

### Empirical data fitting

The 2D models can be fit to simple spatial WM tasks (e.g., Pertzov et al., 2012, 2013) but can also be used for more complex tasks to compare between multiple conditions. Here we fit the models to data collected from people performing a spatial ignore/update WM task (see “Human data” section for details) to show how the models can be useful for more complex experimental designs too.

#### People show biases and nonuniform guesses

To see which model fit the data best, we fit several models to the behavioral data (all conditions). First, we fit the misbinding model and the standard mixture model (no misbinding), and the misbinding model fit best (BIC: 2,984 vs. 3,051). Next we looked at whether the bias functions improved the fit by including each bias in the misbinding model. The constant bias did not improve the fit (BIC = 2,984), while the radial bias did (BIC = 2,966). Adding response sampling improved the misbinding model (BIC = 2,974), as did combining the misbinding model with the radial bias (BIC = 2,958). Therefore, the misbinding model with a radial bias and response sampling was the best-fitting model. The parameters from this fitting are shown in the Supplementary Materials and Supplementary Figure S7 and show that most participants were biased away from the screen center and that these biases were consistent within people across conditions (Pearson correlations: *ρ* > .65, *p* < 0.0001).

#### Convergent validity of parameter recovery

**Figure 14.**
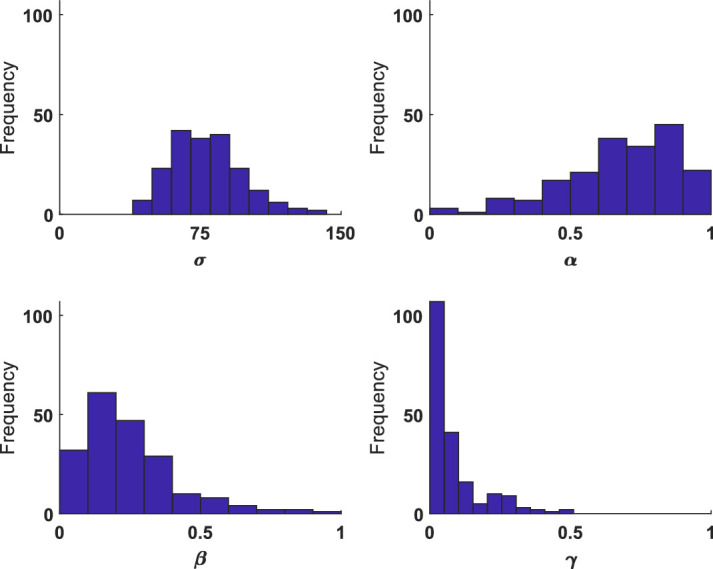
Histograms of the recovered parameters from the ignore/update task, across all conditions.

To compare convergent validity between the 1D and 2D modeling approaches, we compared the fitted parameters from the 2D misbinding model to those reported on the 1D version of this task (Fallon et al., 2018). Histograms of the recovered parameters are shown in Figure 14 and show that while imprecision is approximately normally distributed, the other parameters are skewed, being bounded by 0 and 1. We did not logit transform the α, β, and γ parameters for the comparison to the 1D task as the 1D analysis did not transform parameters (Fallon et al., 2018).

**Figure 15.**
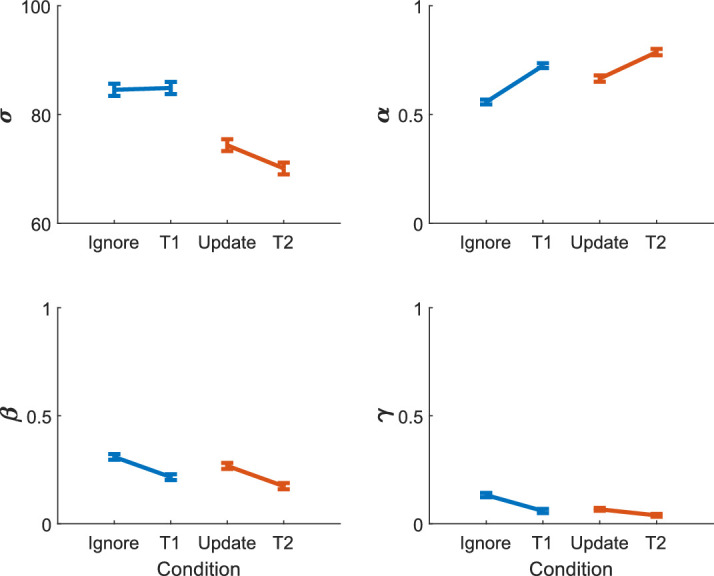
Mean fitted parameter values for each trial type from the healthy participants. Delay increased imprecision (σ; top left; blue vs. orange; see Figure 2 for task explanation), guessing (γ), and misbinding (β; see text for stats); irrelevant stimuli increased misbinding and guessing but not imprecision (ignore and update vs. T1 and T2); and there was an interaction of delay and irrelevant stimuli only for guessing. Standard error bars.

Repeated-measures analysis of variance (ANOVA) with delay and irrelevant stimuli as factors and a random effect of participant were used to analyze the fitted parameters (Figure 15). The analyses showed the following:

■ Delay duration increased the imprecision (*F*(1, 144) = 53.7751, *p* = 1.4959 × 10^−11^), guessing (*F*(1, 144) = 14.4042, *p* = 2.1660 × 10^−4^), and misbinding (*F*(1, 144) = 3.9560, *p* = 0.0486).
■ Irrelevant stimuli increased misbinding (*F*(1, 144) = 19.9033, *p* = 1.6302 × 10^−5^) and guessing (*F*(1, 144) = 19.8437, *p* = 1.6755 × 10^−5^) but did not affect imprecision (*F*(1, 144) = 1.3353, *p* = 0.2498).
■ There was a significant interaction of delay and irrelevant information for guessing (*F*(1, 144) = 4.0163, *p* = 0.0469), although it was only weakly significant and was due to greater guessing in the ignore condition.
■ There were no other interactions (*p* > .1), suggesting that ignore and update trials both affected imprecision and misbinding similarly.

These main effects are similar to the orientation version of this task, although they differ regarding the interaction of delay and irrelevant information. The 1D version found such an interaction in misbinding (Fallon et al., 2018), while here we found it for guessing; in both tasks, the errors were highest in the ignore condition. Potential reasons for this will be discussed later. The similarity in the rest of these results suggests convergent validity of the 2D modeling technique for real human data.

To establish whether the different results in 1D and 2D tasks are due to differences in the modeling, we examined how well each model can distinguish the parameters for the different conditions. We simulated 1D and 2D versions of the task using the mean recovered parameters of each condition from the 1D task (Fallon et al., 2018) and fit the misbinding models using fmincon and inverted the Hessian matrices to get the covariance between parameters. We then used MATLAB's linear hypothesis test to examine whether the recovered parameters for the different conditions were actually from different distributions of parameters (alternate hypothesis) or from the same distribution (null hypothesis).

We performed 1,000 iterations of this and counted how often the 1D and 2D models returned *p* ≤ .05. If the 2D model is better at distinguishing between parameters due to the lower covariance between them, it will be more likely to return *p* ≤ .05 in these linear hypothesis tests. The 2D model found a significant difference between the conditions more often (mean = 29.1% vs. 19.2%). This suggests that these parameters are less confusable (i.e., more distinguishable) in the 2D model than the 1D model. The 2D model was particularly better than the 1D model for comparing the ignore condition with update and T2 conditions, which could mean that the 1D task confused parameters between these conditions, leading to a different interaction being found in the orientation task.

**Figure 16.**
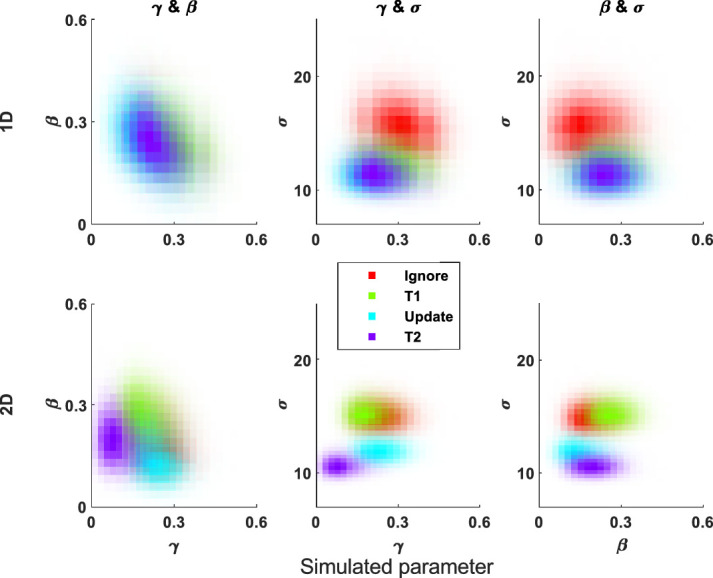
Heatmaps showing the posterior MCMC samples for recovering the mean parameters from Fallon et al. (2018). Each color is a different condition (see legend; Ig = ignore, T1 = long delay, Up = update, T2 = long delay), and the rows show 1D and 2D models, respectively. The γ and β parameters have been arcsine transformed. Both models were simulated using the same parameters. The 2D model has better separation of the different conditions’ parameters, as shown by less overlap between the colors.

To illustrate this, we generated posterior MCMC samples from fitting to data simulated using the mean parameters from the 1D orientation task and plotted the parameters against each other (Figure 16). There was greater separation between the parameters of the different conditions in the 2D model than the 1D model, suggesting that the 2D model is better at distinguishing between parameters of different conditions, within the range recovered from human data.

#### Validating the behavioral metrics

We wanted to see whether the behavioral metrics were actually measuring the types of errors purported. We calculated those behavioral metrics on simulated data and looked at which metrics correlated with the true parameters and compared this against the accuracy of parameter recovery. These simulations used the same stimulus separation constraints as in the “Stimulus separation” section to match the experiments that commonly use these metrics, although running them without such constraints did not change the pattern of results.

We examined several commonly used behavioral metrics. Target distance is the Euclidean distance from responses to targets, while nearest-neighbor distance is the Euclidean distance from responses to their nearest stimulus (Pertzov et al., 2013). The former will contain imprecision, guessing, and misbinding influences, while the latter has attempted to remove the influence of misbinding and thus should contain mainly imprecision and guessing. The difference between these two represents the influence of misbinding. A further measure to capture misbinding, termed *swap errors* (Pertzov et al., 2012), is the proportion of responses occurring within a certain fixed distance (1.5 visual degrees) of a nontarget location. This measure is also confounded by imprecision since imprecise responses to the target location are more likely to end up close to a nontarget by chance, compared to precise responses. We can correct for this by calculating the proportion of possible locations with that specific distance from the target that would have been classed as “swap errors” and subtracting that. Swap errors can also be calculated using the mean target distance as the threshold, effectively scaling the criterion by a participant's average accuracy. This measure can likewise be corrected for chance in the same way.

We correlated the mean values of each of these metrics for simulated data with each of the true parameters (parameter sweep from previous simulations, 10 iterations). All the metrics correlated with multiple true parameters, so we present here only the metric most strongly correlated with each parameter (see Supplementary Figure S9 and Supplementary Table S1 for full correlation matrix between metrics and parameters).

**Figure 17.**
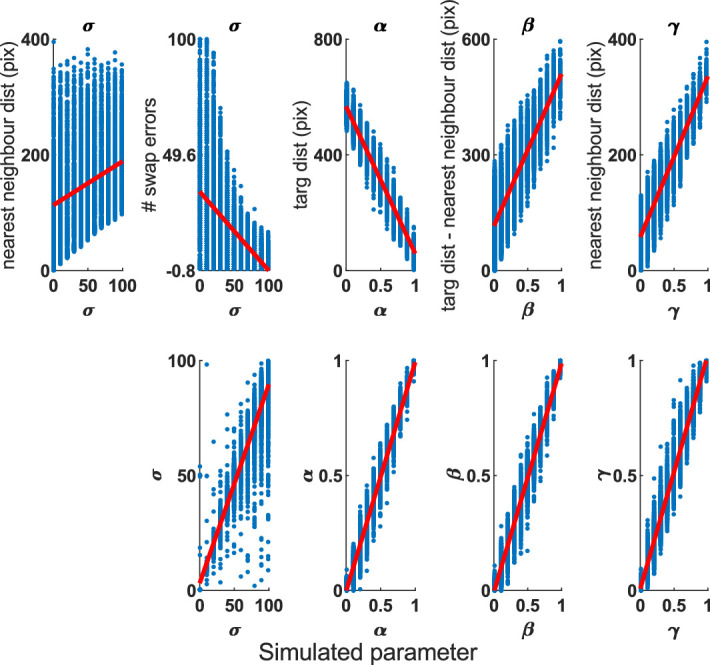
Relationship between the true parameters (x-axis) and the best behavioral metrics (top) and recovered parameters (bottom). All correlations were significant (*p* < 10^−10^). Two metrics were compared against the imprecision (σ) parameter. The recovered parameters had stronger correlations than the best behavioral metrics (see text for statistics).

Target distance correlated strongly and negatively with the proportion of target responding (Figure 17; Spearman correlations: *r* = –.9687, *p* < 10^−10^), nearest-neighbor distance positively with guess rate (*r* = .9279, *p* < 10^−10^), and the difference between these positively with misbinding (*r* = .8589, *p* < 10^−10^). There were no metrics that correlated strongly with imprecision, although swap errors (fixed threshold) had a medium negative correlation (*r* = –.5519, *p* < 10^−10^) while the nearest-neighbor distance (assumed to be a good measure of imprecision) had a weak positive correlation with it (*r* = .2931, *p* < 10^−10^), so both of these are presented. These metrics were all less strongly correlated with the true parameters than the recovered parameters were, although this may be an artifact of recovering the parameters with the same model used for simulation, as this makes it unlikely the behavioral metrics can perform better.

To investigate this idea, we compared the split-half reliability of these four behavioral metrics and the recovered parameters for these behavioral data (pooling across all four conditions, 100 iterations of random splitting). All measures showed significant split-half reliability (Spearman correlations: *p* < 0.0001) with varying strengths. The swap errors metric was only weakly correlated with itself (*ρ* = 0.2068), while the imprecision parameter was strongly correlated (*ρ* = 0.7160). Target distance was slightly more reliable (*ρ* = 0.7955) than the recovered α parameter (*ρ* = 0.7811), but nearest-neighbor distance (*ρ* = 0.8196) and target minus nearest-neighbor distance (*ρ* = 0.7450) were better than γ (*ρ* = 0.3347) and β (*ρ* = 0.6278). This suggests that some of the behavioral metrics have good split-half reliability, and these are the ones that are most strongly correlated with the true α, β, and γ parameters in the simulations above. However, these simulations and correlations suggest none of the behavioral metrics currently used are good estimators of memory imprecision, and the ones currently available have low reliability. They also suggest that guessing and misbinding estimation in the model has poor split-half reliability, which could be due to the low guessing and misbinding parameters used for these simulations (recovered from the behavioral data), meaning few trials contain a guess or misbind, which leads to poorer estimation in the split-halves.

We can also look at convergent validity by comparing the outputs of ANOVA on the behavioral metrics in the 2D human data to those previously reported on the 1D circular analogue task, as we did earlier with the 2D modeling outputs. These showed that delay increased target distance (*p* < 0.0001), nearest-neighbor distance (*p* < 0.0001), and the difference between these two (*p* = 0.0077), while irrelevant distractors increased swap errors (*p* = 0.0001), target distance (*p* < 0.0001), and target minus nearest-neighbor distance (*p* < 0.0001)—but not the nearest-neighbor distance itself (*p* = 0.6066). There were no interactions (*p* > 0.1). While target distance and target minus nearest-neighbor distance patterns are similar to α and β (in the 1D data and our 2D data), the nearest-neighbor distance effects (delay but not irrelevance) were more similar to that seen in imprecision than guessing. This may not be surprising as the correlations (Figure 17) showed nearest-neighbor distance was correlated with both imprecision and guessing. This suggests that while nearest-neighbor distance is reliable, it is not a pure measure of one type of error source.

## Discussion

We have developed a 2D mixture modeling toolbox for tasks such as spatial WM tasks. The models are able to recover parameters from simulations with good accuracy across a large parameter space (Figure 3) in line with the standard 1D mixture models and have less interdependent parameters (Figure 4). The accuracy rises as the number of trials increases (Figure 5) or the number of nontargets decreases (Figure 6). Behavioral data from a complex spatial WM task showed a similar pattern of results to a 1D orientation analogue of the 2D task, giving convergent validity (Figure 15). The 2D modeling provided a better estimate of true parameters of simulations than commonly used behavioral metrics (Figure 17). This suggests that the 2D mixture modeling approach is valid for analysis of data from complex spatial WM tasks and is more sensitive than previously available behavioral metrics. The models are provided in the MemToolbox2D package (Grogan et al., 2019; https://doi.org/10.5281/zenodo.3752705), compatible with the MemToolbox package for modeling 1D tasks (Suchow et al., 2013; MemToolbox.org).

The fact that the 2D models outperform 1D models in the simulations can be understood when examining the correlations of the different parameters from the posterior samples (Figure 4). The 2D model's parameters are less strongly (albeit still significantly) associated with each other than the 1D model's parameters. Mixture modeling can give accurate and reliable estimation of parameters in 1D tasks, helped by the fact that responses are often tightly clustered around stimuli locations (i.e., low imprecision). However, as imprecision increases, responses become more and more likely to land near other stimuli and be classed as misbinds; due to the circular space, if you move far enough away from a target, you will hit a nontarget. In the 2D task, while increasing imprecision does increase the chance of hitting a nontarget, this is overall much lower as there are *two* uncorrelated dimensions of separation between stimuli, and the responses do not wrap around the screen edges, instead being contained by them. This means that you are not guaranteed to hit a nontarget as you move further from a target, unlike in the circular task—sometimes you will hit a screen edge instead. These factors mean that the 2D models are better able to distinguish imprecision, guessing, and misbinding; these differences are due to differences in the task setup rather than the models themselves.

The simulations presented here used uniformly random item locations and performed well, but adding in stimulus constraints such as minimum distances to other items, screen edges, and screen center improved the parameter recovery, particularly for guessing and misbinding. Such constraints mean that the probability density functions for target and misbinding responses have less overlap and are cut off less by the screen edges, leading to better estimation of the probabilities of each type of response. This analysis also demonstrates how important simulation can be in task design, allowing the experimenter to try out different stimulus constraints, number of trials, and numbers of nontargets to optimize their task for the recovery of parameters of interest.

We applied the models developed here to behavioral data from healthy older adults on a complex ignore/update spatial WM task, a spatial version of a previously used orientation task (Fallon et al., 2018). The misbinding model fit the data well and analysis of the parameters revealed that delays increased imprecision, guessing, and misbinding, while irrelevant stimuli increased guessing and misbinding but not imprecision. This pattern is similar to that reported in the 1D orientation version, although here we found an interaction of delay and irrelevant stimuli on guessing—not misbinding as previously reported for the orientation WM task (Fallon et al., 2018). In that paradigm, there was much more misbinding when participants had to ignore distracting stimuli than when they had to update the items in WM or simply maintain them over delays, whereas in our 2D data, this pattern was observed for guessing. This difference could be due to methodological differences, such as the smaller sample with older participants used here (older adults have worse WM; McNab et al., 2015; Pertzov, Heider, Liang, & Husain, 2015), or other differences in the task such as the minimum stimulus distance constraints in the 2D task. Importantly, before the location-memory test, the participants had to choose which of two items was a target (the other being a novel foil or a distractor from that trial); this stage was not present in the 1D version of the task and could have contributed to the differences here. However, analyzing only those trials that were correctly identified at this stage did not change the pattern of results recovered (see Supplementary Figure S8).

Alternatively, the 2D modeling was shown to be more sensitive during simulations and to have less interdependent parameters (i.e., weaker correlations between recovered parameters) than the 1D modeling, and simulations from the fitted parameters showed the 2D model was better at distinguishing between parameters from different conditions. The different pattern of responses could therefore be due to more accurate parameter recovery in 2D tasks. Finally, and most interestingly, it is possible that the different results reflect differences between the way locations and orientations are processed in WM. There is a proposal that location plays a fundamentally distinct role in WM, such that all other features of an item (e.g., orientation, color, shape) are bound to location (Schneegans & Bays, 2017). It is possible that orientations are misbound more when distracting information is to be ignored, but locations are not as they are the feature to which others are bound.

Other spatial WM tasks have reported an interaction of delay and number of items (Pertzov et al., 2012), which was present in the overall target distance but not in the nearest-neighbor distance (i.e., when controlling for misbinding). This was taken to mean that larger memory loads for longer durations lead to more misbinding errors but did not affect the imprecision of the memory. These behavioral metrics have been used in many spatial WM tasks (Liang et al., 2016; Pertzov et al., 2013; Zokaei, Nour, et al., 2019) and the current modeling approach provides a way to examine the validity of those metrics for estimating different sources of error.

Comparing the true parameters of simulations against traditional behavioral measures for this task gave a sensible pattern of results. Mean target distance was associated with imprecision, guessing, and misbinding. Nearest-neighbor distance (which removes the influence of misbinding to give a better estimate of imprecision) had no association with misbinding but instead associated mainly with guessing and a little with imprecision. The difference between these two was a good estimate of misbinding. These relationships were all expected and assumed in previous studies relying on them (Pertzov et al., 2012, 2013).

However, imprecision was not strongly associated with any behavioral metric, suggesting it independently quantifies a new aspect of performance that is not well captured by existing measures. Imprecision had medium and weak associations with the proportion of swap errors and nearest-neighbor distance, respectively. The behavioral measures were less strongly correlated with “true” parameters used for simulations than recovered parameters were, but this is not surprising as the same model was used for simulation and fitting. While the simulations here show that the models can accurately recover simulated parameters, people likely use a range of behavioral heuristics not captured by the models and functions provided here. Split-half reliability and convergent validity checks showed that while target selection and imprecision could be reliably measured by the metrics, the metrics associated with misbinding and guessing were less reliable and valid measures.

Two-dimensional mixture modeling has been used previously (Schneegans & Bays, 2016) on a task in which stimuli appeared within an annulus of 5° width, thus having two dimensions, in contrast to tasks where stimuli locations appear on a ring (i.e., with no width) (Rajsic & Wilson, 2014; Schneegans & Bays, 2018), meaning the 2D coordinate data can be treated as a 1D orientation and modeled with the existing 1D model toolboxes (Suchow, Brady, Fougnie, & Alvarez, 2013). To account for this spatial constraint, the authors built in priors to their model, such that the bivariate normal distributions around targets and nontargets and the uniform distribution were convolved with an estimated distribution of their response eccentricities (which closely matched the annulus location and width). This method illustrates the flexibility of mixture modeling for 2D tasks with stimuli or response constraints, as well as the usefulness of incorporating priors into the model. This method also bears similarity to the response sampling method presented here, although applied to not only the guessing distribution but also the target and nontarget distributions, which could be useful for future researchers—the toolbox provided here is adaptable, and an example model outline is provided to show how new or modified models may be created.

There are some limitations with adapting the models to two dimensions. Previous 1D tasks often use a circular space (e.g., orientation or color), and this unbounded space works well because of the lack of edge effects. In contrast, 2D tasks using screens for stimuli presentation have a bounded space, meaning that responses do not “wrap around” as they do in a circular space. In this case, assumptions must be made regarding behavior close to the boundary and in the generative models that may place items outside of the boundaries. The generative models implemented here are likely not the true process that occurs when people recall items near the edge of the display, but it seems a reasonable simplification that still produces a well-fitting model.

In reality, people may have repelling biases away from edges or anchoring or landmark effects close to them. The bias functions in the MemToolbox2D package can test for some of these, and more complicated models can be developed that include these, and other, effects. We also provide a “response sampling” method that builds a response density function over all the responses from a person and can be used to sample the guesses from. This is an alternative to assuming a uniform guessing distribution. There are myriad potential biases and guessing distributions, and the MemToolbox2D package is provided with a permissive license to allow modification of the code here, as was the original MemToolbox package (Suchow et al., 2013).

While these models were designed for WM tasks, they can be used for other types of tasks, including long-term memory tasks for spatial locations (e.g., Zokaei, Čepukaitytė, et al., 2019). There is also no reason why the models cannot be extended into the third dimension, should experiments require it, for example, in virtual reality navigation tasks.

MemToolbox2D is provided as free code available from https://doi.org/10.5281/zenodo.3752705 (Grogan et al., 2019).

## Conclusion

We provide here a new toolbox (MemToolbox2D; Grogan et al., 2019; https://doi.org/10.5281/zenodo.3752705) for analyzing 2D spatial WM tasks. Simulations showed it is accurate at recovering parameters over a wide range of values, number of trials, and number of nontargets and can take into account response distributions and biases. It provides a more accurate measure of true parameter values than previously used behavioral metrics on such tasks and may be better at separating out codependent parameter values because of the second dimension of separation present in the data.

## Supplementary Material

Supplement 1
