# Supplementary material for: A new toolbox to distinguish the sources of spatial memory error
Source: J Vis. 2020 Dec 8;20(13):6. doi: 10.1167/jov.20.13.6 (PMC7726590; doi:10.1167/jov.20.13.6)
Supplement: Supplement 1 [file jovi-20-13-6_s001.pdf]

# Supplementary Materials: A new toolbox to distinguish the sources of spatial memory error

## Model parameter recovery

The following figures show the parameter recovery errors across a wide range of imprecision, guessing and misbinding parameters for the 2D misbinding model. As the guessing and misbinding parameters must sum to less than 1, those parameter values are truncated in the presented plots. The same figures for the 1D model are shown in the Supplementary Materials, showing a similar pattern, but poorer parameter recovery across the parameter space. In summary, high levels of guessing preclude accurate recovery of precision but not misbinding, whereas high levels of misbinding do not impact much on the other parameters, and high imprecision can result in overestimation of guessing and underestimation of misbinding.

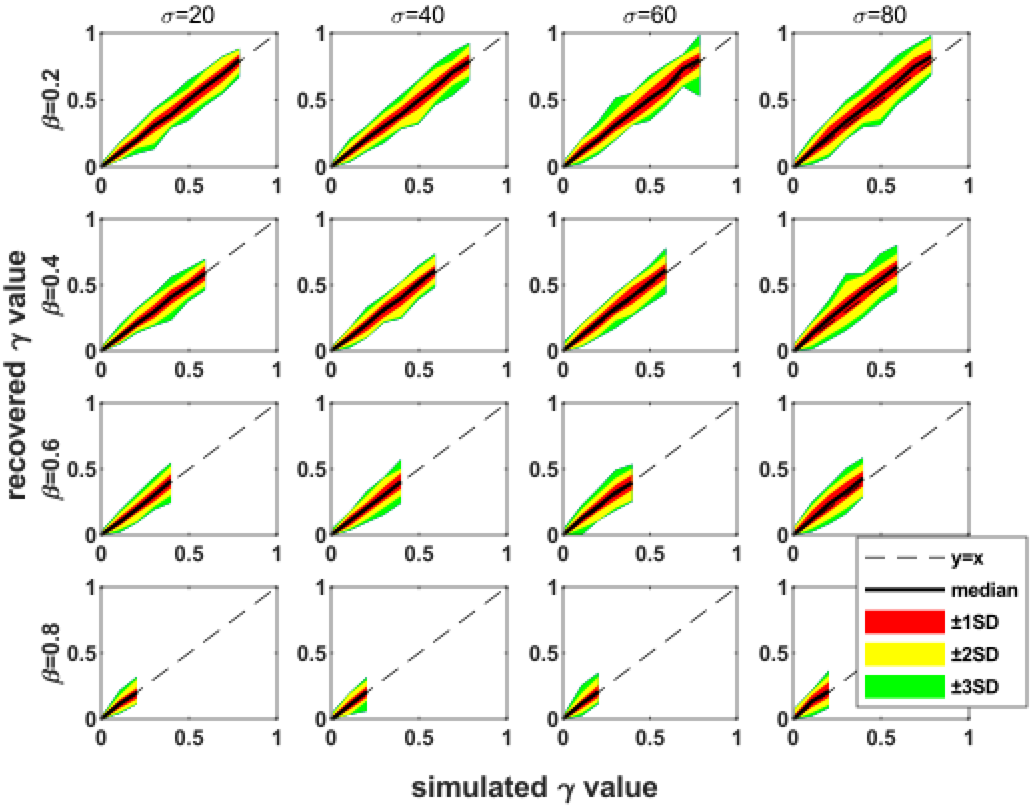


***Figure S1.*** *Simulated and recovered guessing parameters (γ) for slices taken through the parameter sweep.*

*Each box shows the median (solid black line) recovered guessing parameter values (± standard deviations – see legend) plotted against the true guessing parameters, for a different combination of misbinding and imprecision parameters. The columns show increasing imprecision, and the rows show increasing misbinding. As imprecision increases, guessing is overestimated, but increasing misbinding has little effect. The values are truncated because $\beta + \gamma$ cannot exceed 1. The dotted line shows y=x.*

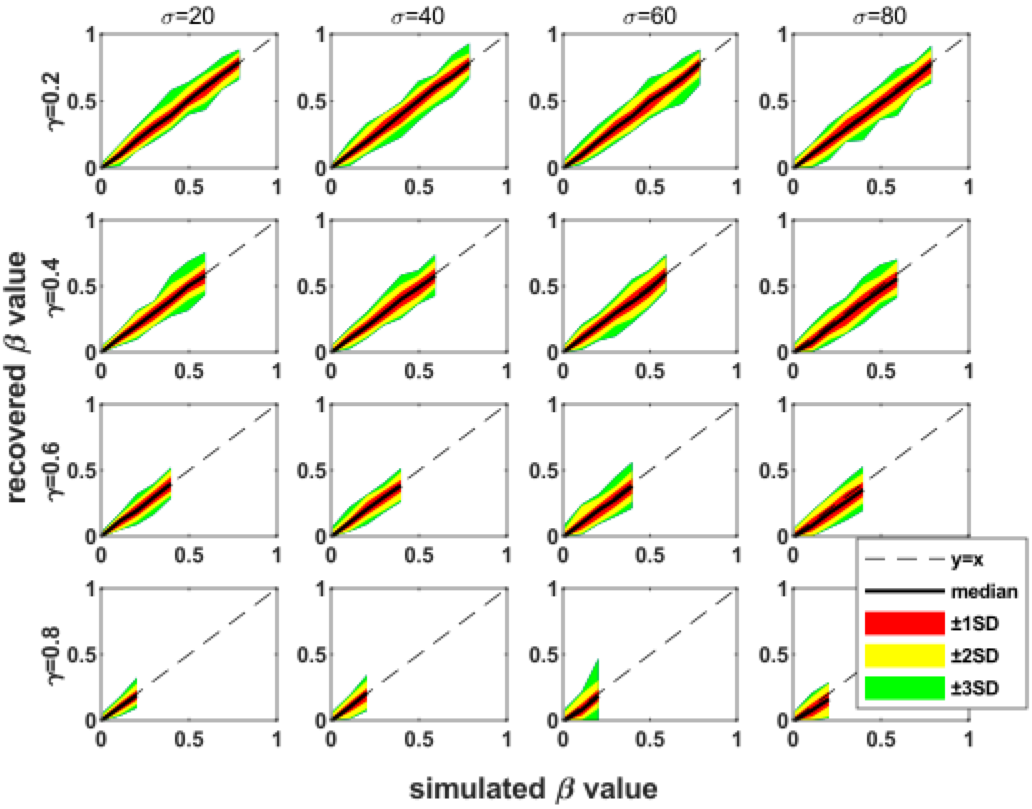


***Figure S2.*** *Simulated and recovered misbinding parameters (β) for slices through the parameter sweep.*

*Each box shows the median (solid black line) recovered misbinding parameter values (± standard deviation: shading) plotted against the true misbinding parameters, for a different combination of guessing (rows) and imprecision (columns) parameters. As imprecision increases, misbinding is underestimated, but increasing guessing has little effect. The values are truncated because β + γ cannot exceed 1. The dotted line shows y=x.*

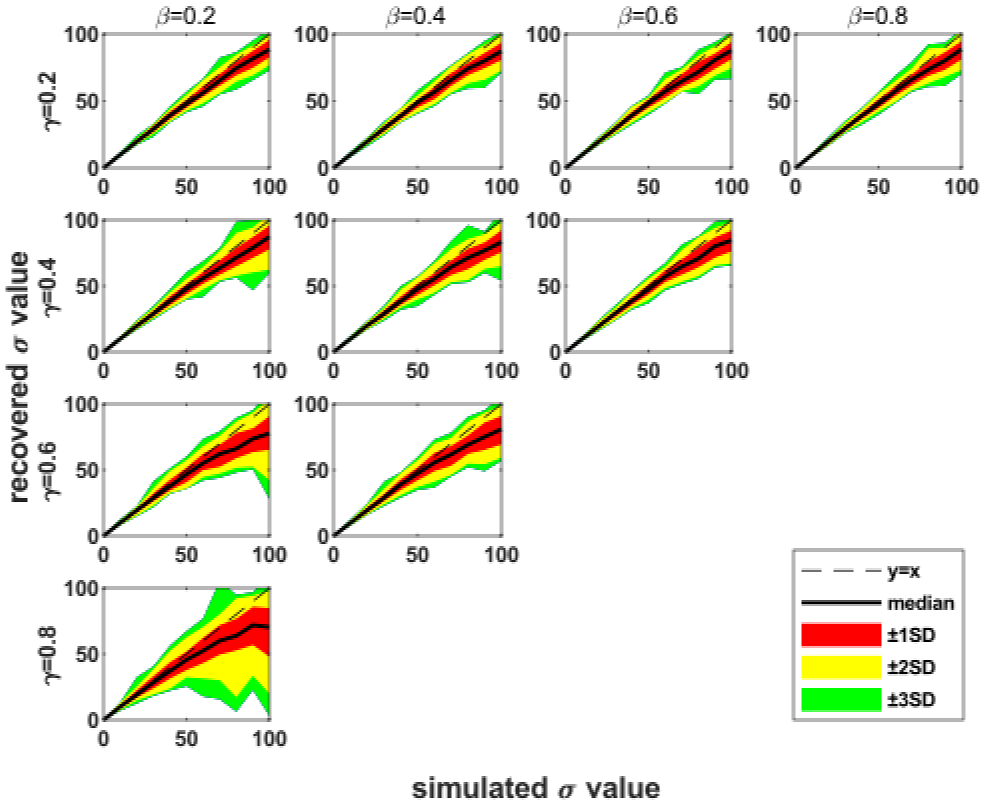


***Figure S3.*** *Simulated and recovered imprecision parameters (SD, σ) for slices through the parameter sweep.*

*Each box shows the median (solid black line) recovered imprecision parameter values (± standard deviation: shading) plotted against the true imprecision parameters, for a different combination of misbinding (columns) and guessing (rows) parameters. As guessing increases, large imprecision values are underestimated, while increasing misbinding has little effect. The dotted line shows y=x.*

We also simulated and fit the 1D misbinding model across the same parameter sweep as in the 2D model presented above, for comparison.

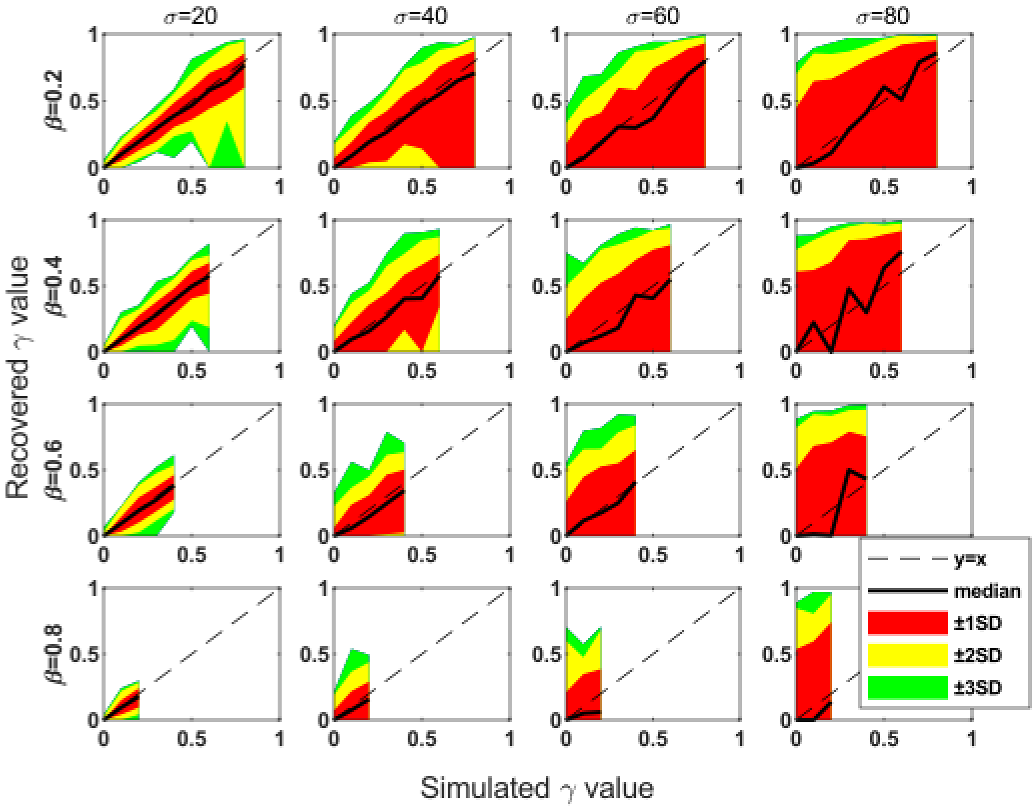


***Figure S4.*** *The simulated and recovered guessing parameters (γ) for the 1D misbinding model for slices taken through the parameter sweep.*

*Each box shows the mean (solid black line) recovered guessing parameter values (± standard deviations – see legend) plotted against the true guessing parameters, for a different combination of misbinding and imprecision parameters. The median values are accurate, but the variance is much larger than the 2D model (**Figure S1**). The columns show increasing imprecision, and the rows show increasing misbinding. The values are truncated because β + γ cannot exceed 1. The dotted line shows y=x.*

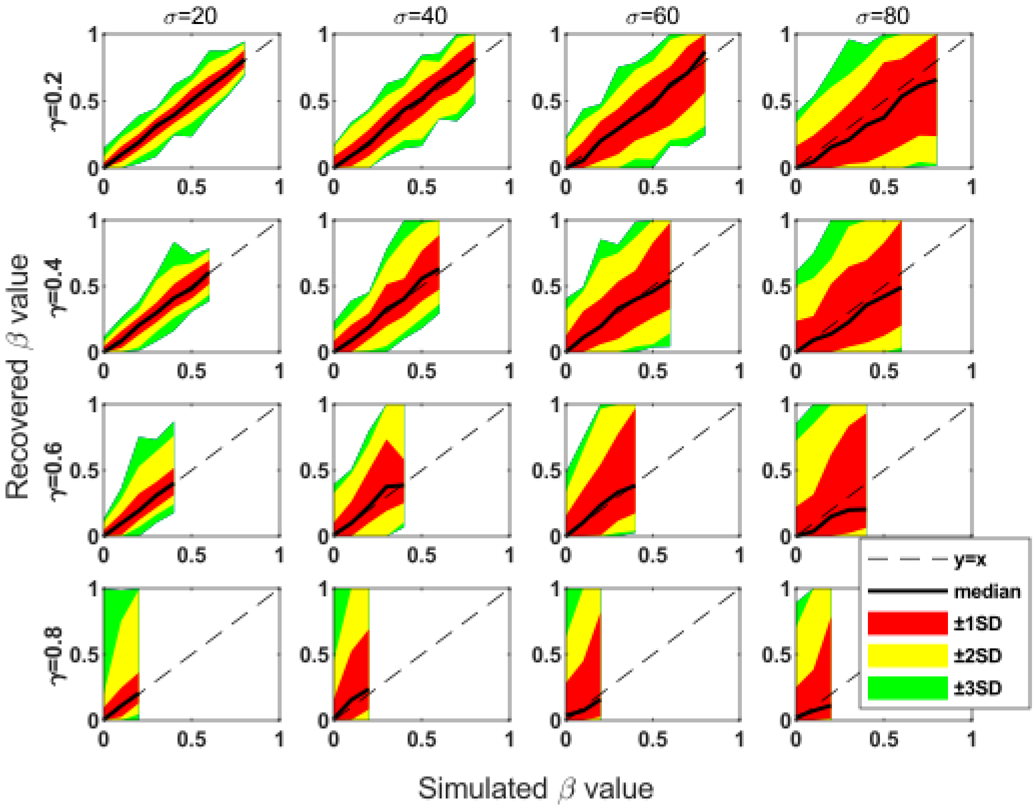


***Figure S5.*** *The simulated and recovered misbinding parameters (β) for the 1D misbinding model, for several slices through the parameter sweep.*

*Each box shows the mean (solid black line) recovered misbinding parameter values (± standard deviations – see legend) plotted against the true misbinding parameters, for a different combination of guessing (rows) and imprecision (columns) parameters. As imprecision increases, misbinding is underestimated, and the variance of the recovered parameters increases with imprecision and guessing values. The values are truncated because β + γ cannot exceed 1. The dotted line shows y=x.*

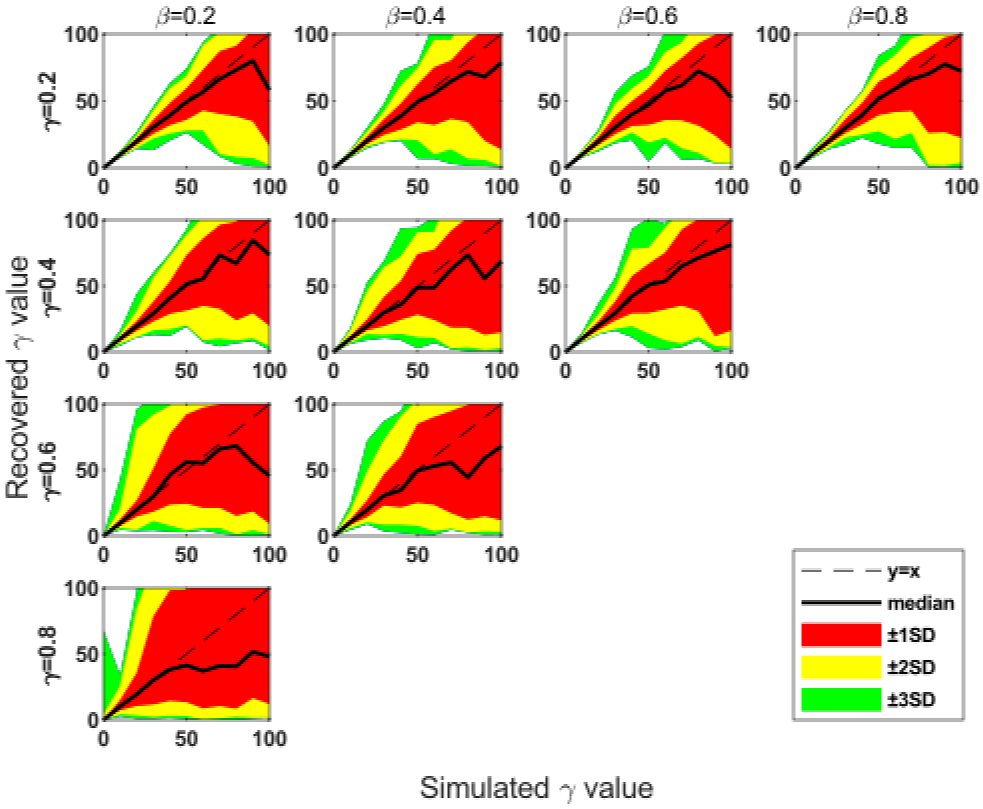


***Figure S6.*** *The simulated and recovered concentration parameters (κ) for the 1D misbinding model, for several slices through the parameter sweep.*

*Each box shows the mean (solid black line) recovered imprecision parameter values (± standard deviations – see legend) plotted against the true imprecision parameters, for a different combination of misbinding (columns) and guessing (rows) parameters. As guessing increases, imprecision is underestimated, while misbinding has little effect. The dotted line shows y=x.*

## Model Comparison

We fit several models to the behavioural data, and found the best fitting one was the misbinding model with a radial bias and response sampling for the guessing distribution. In the main text, we analysed the parameters of the misbinding model (without radial bias or response sampling) as we were comparing against an earlier paper that used the misbinding model in a 1D tasks. Here we analyse the parameters from the best fitting model.

Delay increased imprecision ($F$ (1, 144) = 33.7362, $p$ = 3.8833x10$^{-8}$), guessing ($F$ (1,144) = 21.3417, $p$ = 8.4523x10$^{-6}$), but not misbinding ($F$ (1, 144) = 0.5449, $p$ = .4616). Irrelevant information increased misbinding ($F$ (1, 144) = 11.1125, $p$ = .0011) and guessing ($F$ (1, 144) = 20.8149, $p$ = 1.0742x10$^{-5}$) but not imprecision ($F$ (1, 144) = 0.4771, $p$ = .4908). There was a borderline significant interaction of delay and irrelevant stimuli on guessing ($F$ (1, 144) = 3.8905, p = .0505), again due to higher guessing in the ignore condition.

The radial bias parameter had an effect of irrelevant information (F (1, 144) = 6.7668, *p* = .0103) and an interaction of delay and irrelevant (*F* (1, 144) = 7.0429, *p* = .0089), both driven by higher (less negative) biases for the T2 (short delay) condition. This means the short delay condition had less of a bias towards the centre of the screen than the other conditions.

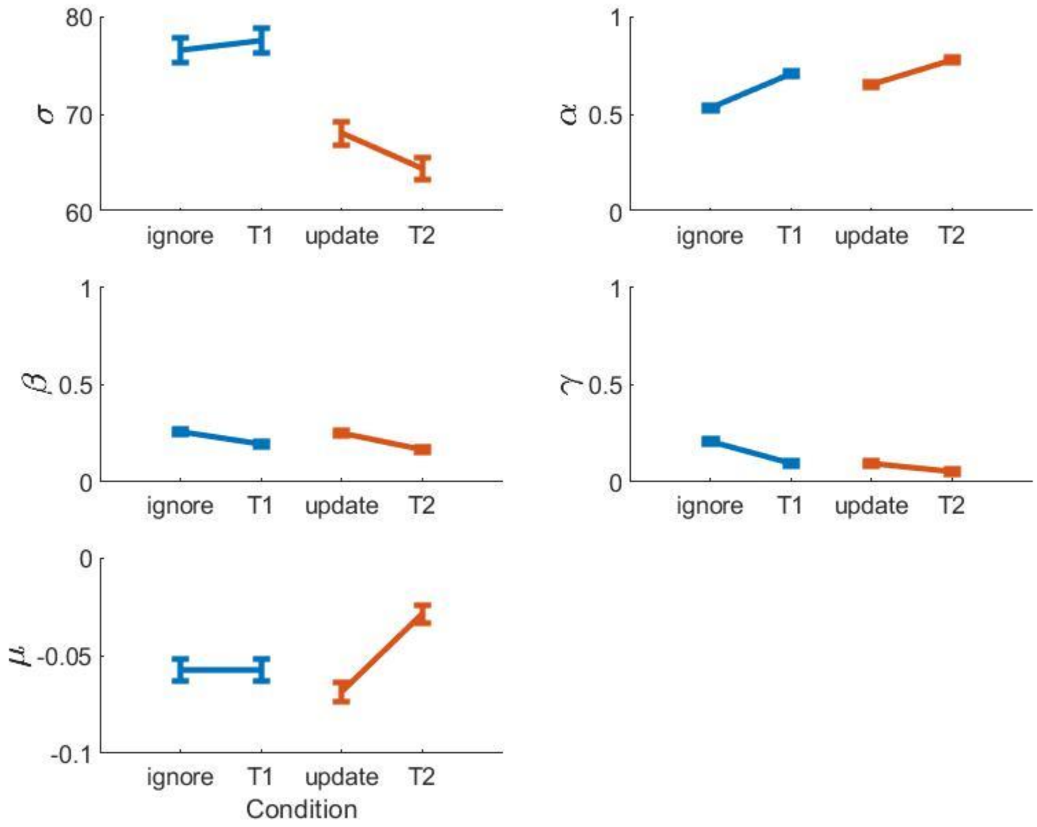


***Figure S7.*** *The fitted parameters from the misbinding model with radial bias (μ) and response sampling. Standard error bars. See text for stats.*

*The radial bias parameters showed greater correlations within subjects (across conditions) than the other parameters, suggesting it is fairly stable across these manipulations.*

## Identification accuracy

As the 2D task analysed here had an extra stage not present in the 1D versions, where two items were presented and participants chose the target item and then reported the location of it, we re-analysed our data after excluding those trials where participants chose the wrong item (18% of trials on average).

The misbinding model returned similar results, with significant effects of delay for imprecision (*F* (1, 144) = 39.598, *p* = 3.5362x$10^{-9}$), target selection (*F* (1, 144) = 18.844, *p* = 2.6579x$10^{-5}$), misbinding (*F* (1, 144) = 5.6393, *p* = .018881) and guessing (*F* (1, 144) = 19.949, *p* = .0011831). Irrelevant stimuli

affected target responding ($F$ (1, 144) = 42.592, $p$ = 1.0741x10$^{-9}$), misbinding ($F$ (1, 144) = 19.156, $p$ = 2.3004x10$^{-5}$) and guessing ($F$ (1, 144), $p$ = .00033567). There was a significant interaction of delay and irrelevant stimuli for imprecision ($F$ (1, 144) = 5.932, $p$ = .016091), target selection ($F$ (1, 144) = 4.0495, $p$ = .046048) and guessing ($F$ (1, 144) = 5.4748, $p$ = .020666) but not misbinding ($F$ (1, 144) = 0.36127, $p$ = .54875).

The main difference was on imprecision, as now there was an interaction such that irrelevant information increased imprecision for short delays (update vs T2) but slightly decreased it for long delays (ignore vs T1). The interactions were unchanged for guessing and misbinding, suggesting that the difference between 1D and 2D tasks may not have been due to differences in this target-identification stage.

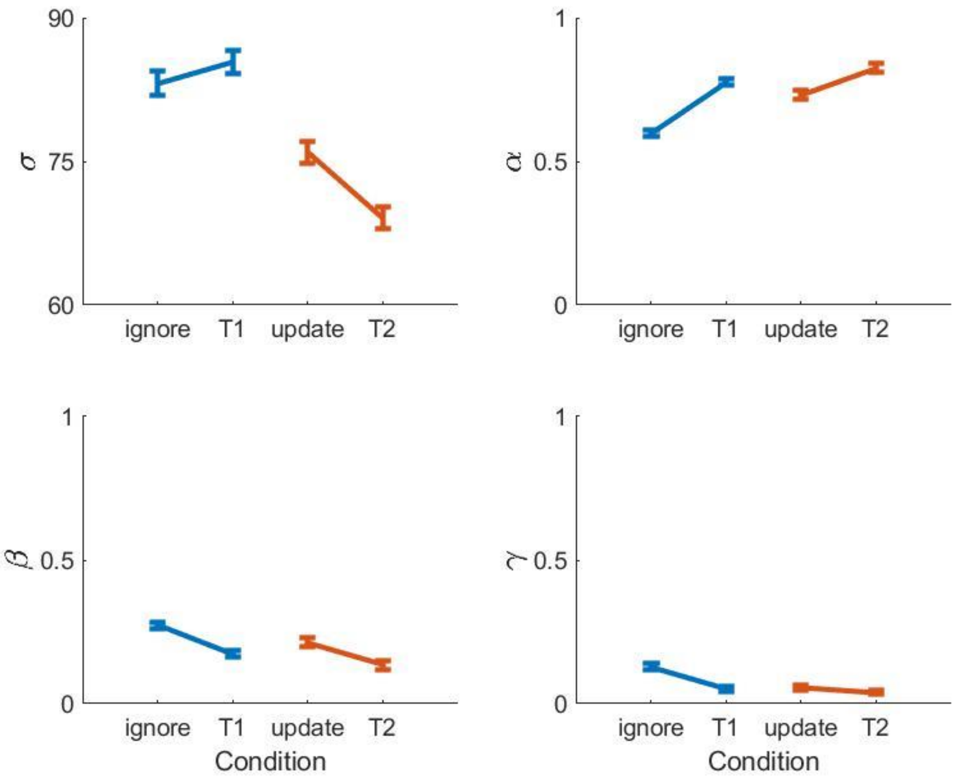


***Figure S8.*** *Mean parameters from misbinding model fit for correctly identified targets only. Standard error bars.*

## Behavioural Metrics Correlations

The correlation of each behavioural metric and each true parameter from the simulations is show here. Interestingly, the swap error metrics were not the best associated with the misbinding parameter, but the swap errors using mean target distance as the threshold did come close, albeit when uncorrected for chance.

***Table S1.*** *Spearman's correlation coefficients between true parameters for simulations and the different behavioural metrics. This is from simulations across full parameter sweep, with 10 iterations. The presented metric(s) presented for each parameter are indicated by †. All correlations were significant to* $p < 10^{-10}$.

| | *σ* | *α* | *β* | *γ* |
|---|---|---|---|---|
| Target Distance | 0.0838 | -0.9687† | 0.4822 | 0.4865 |
| Nearest-neighbour Distance | 0.2931† | -0.4638 | -0.4641 | 0.9279† |
| Target Distance – Nearest-neighbour Distance | -0.0961 | -0.8088 | 0.8589† | -0.0501 |
| Proportion of Swap Errors | -0.5519† | -0.3274 | 0.6469 | -0.3195 |
| Swap Errors Corrected | -0.5503 | -0.3301 | 0.6491 | -0.3190 |
| Swap Errors (Mean Threshold) | 0.0287 | -0.8701 | 0.8141 | 0.0561 |
| Swap Errors Corrected (Mean Threshold) | -0.0928 | 0.2284 | 0.1825 | -0.4109 |

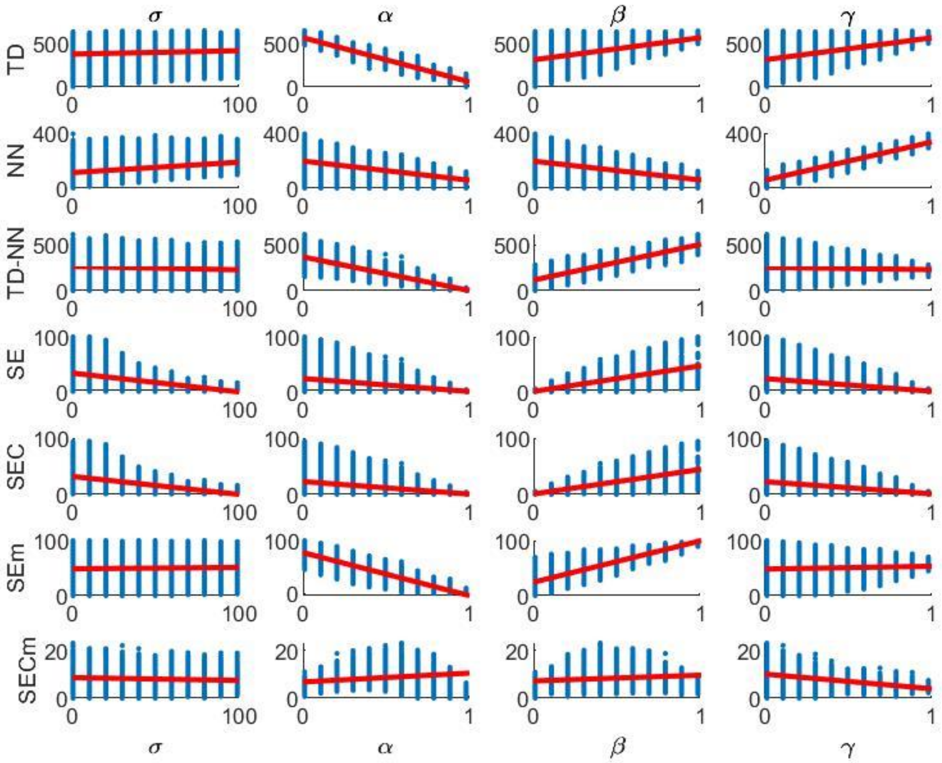


***Figure S9.*** *Scatter plots showing relationship between each behavioural metric and true parameter for model simulations.*

*Columns are the parameters, Rows are the behavioural metrics, in order: Target Distance, Nearest-neighbour Distance, the difference between those two, proportion of Swap Errors, Swap errors corrected, Swap Errors using mean target distance as threshold, Swap Errors (mean distance) corrected. Red lines are linear best-fit lines. The Spearman's correlations are presented in **Table S1**.*

## Change Detection

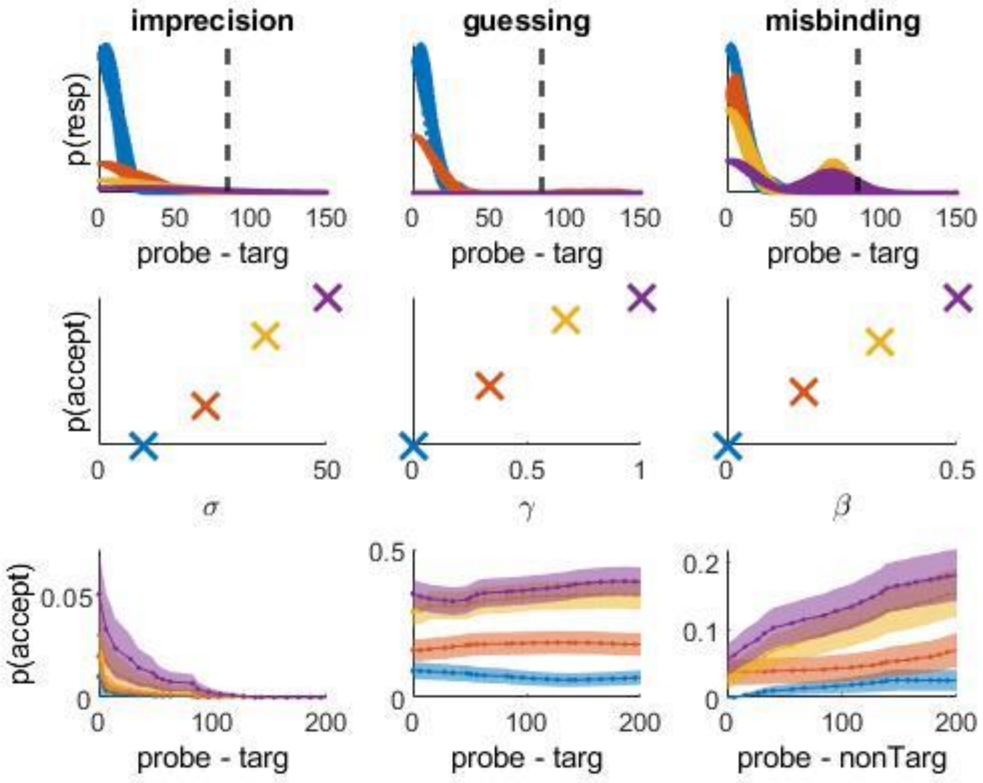


*Figure S10. Illustrative examples of 2D mixture modelling approach to change detection.*

*We show 2D examples to match the 1D examples shown in **Figure 12**. The top row shows how each error component affects the proportion of generative responses that would fall at or beyond a probed distance of 60 pixels (arbitrary y-units). The second row shows the values of the parameters used for those simulations, along with the summed probabilities of all responses which occur at >= 60 pixels from the target. Increasing each parameter increases the probability of incorrectly accepting this probe as unchanged. The bottom row shows the probability of accepting a probe as unchanged as a function of the probe distance (or probe-non-target distance for misbinding). This shows that increasing imprecision increases the spread of these same responses around the target, increasing guessing affects all orientations similarly, and increasing misbinding affects responses in relation to their distance to the distractor (if we plotted probe-targ for misbinding, it would look like the guessing panel).*
